# Supplementary material for: NEUROlogical Prognosis After Cardiac Arrest in Kids (NEUROPACK) study: protocol for a prospective multicentre clinical prediction model derivation and validation study in children after cardiac arrest
Source: BMJ Open. 2020 Sep 25;10(9):e037517. doi: 10.1136/bmjopen-2020-037517 (PMC7520830; doi:10.1136/bmjopen-2020-037517)
Supplement: Supplementary data [file bmjopen-2020-037517supp001.pdf]

PICANet Custom Audit Definitions

NET-PACK 3

Version 1.1 (May 2017)

Contents

Introduction 2

- Background 2
- References 2
- Data collection method 3

Patient details 6

- Family name or Surname 6
- First name 6
- Postcode 6
- NHS, CHI or H&C number 7
- Case note number 7
- Date of birth 7

History at admission 8

- Bystander Cardiopulmonary Resuscitation (CPR) Attempted? 8
- Cardiopulmonary Resuscitation continued after arrival to the Emergency Department? 8
- First monitored cardiac rhythm during cardiac arrest 9
- Time from observed cardiac arrest to start of sustained return of spontaneous circulation (ROSC) 9
- Number of doses of epinephrine from initial resuscitation to start of period of sustained ROSC... 10

Temperature management 10

- Core body temperature management planned during first 24 hours after sustained ROSC 10
- Duration of initial active temperature control management 11
- Minimum temperature recorded during first 24 hours 11
- Maximum temperature recorded during first 24 hours 11

Comments 12

Form completed by 12

Scholefield BR, *et al. BMJ Open* 2020; 10:e037517. doi: 10.1136/bmjopen-2020-037517

Introduction

Background

The NET-PACK 3 Custom Audit - PICANet evaluation of Post cardiac Arrest Care in Kids, is a re-audit of patient management after cardiac arrest in UK and Irish PICUs.

Between June 2014 and December 2015, in collaboration with Dr Barney Scholefield (Chief Investigator) at Birmingham Children's Hospital PICU and the Paediatric Intensive Care Society (PICS), PICANet performed the NET-PACK **2** custom audit in 29 UK and Irish PICUs.

Additional data was collected about post cardiac arrest management for either out-of-hospital or in-hospital cardiac arrest prior to PICU admission in 400 infants and children. Eight resuscitation variables available at the time of PICU admission and the early proposed post cardiac arrest temperature management plans were collected. The key findings will be published in detail shortly.

Importantly wide variation in PICU post-arrest management has been identified and also opportunities to stratify the cardiac arrest population for targeted treatments.

NET-PACK 3 has been designed to investigate the impact and compliance with the new International guidance and research data on post-arrest care as part of the PICANet clinical audit function. In December 2015 the International Liaison Committee on Resuscitation (ILCOR) published up-to-date guidance on Paediatric Advanced Life Support and post-cardiac arrest management (1). In addition two large randomised controlled trials of targeted temperature management after paediatric cardiac arrest have been published (2, 3). The primary objective of the NET-PACK 3 custom audit will be to assess whether targeted temperature management (TTM) is used, the dose of TTM (duration and temperature) following the ILCOR 2015 guidance and trial recent publications and the effect on survival outcome. In addition the NET-PACK 3 Custom Audit data will be available for linkage in centres participating in the NIHR funded NEUROdevelopmental Prognositic after Cardiac Arrest in Kids Trial (NEURO-PACK). This trial will be evaluating more detailed neuro-developmental outcomes of patients after paediatric cardiac arrest.

References

1. de Caen AR, Maconochie IK, Aickin R, Atkins DL, Biarent D, Guerguerian AM, et al. Part 6: Pediatric Basic Life Support and Pediatric Advanced Life Support: 2015 International Consensus on Cardiopulmonary Resuscitation and Emergency Cardiovascular Care Science With Treatment Recommendations. Circulation. 2015;132(16 Suppl 1):S177-203. Epub 2015/10/17.
2. Moler FW, Silverstein FS, Holubkov R, Slomine BS, Christensen JR, Nadkarni VM, et al. Therapeutic Hypothermia after In-Hospital Cardiac Arrest in Children. N Engl J Med. 2017;376(4):318-29. Epub 2017/01/25.
3. Moler FW, Holubkov R, Dean JM. Therapeutic Hypothermia in Children. N Engl J Med. 2015;373(10):980. Epub 2015/09/04.

Data collection method

For units who agree to participate in this custom audit PICANet will enable access to the specific custom audit data collection tab on the data entry page:-

1. A PICANet NET-PACK 3 custom audit form (see below) is completed for all admissions for either out-of-hospital or in-hospital cardiac arrest prior to PICU admission.

PICA Net

Paediatric Intensive Care Audit Network · Custom Audit

NET-PACK 3

NET-PACK 3: PICANet evaluation of post cardiac arrest care in kids

Please complete for ***all PIC admissions following cardiac arrest*** *(include both out-of-hospital and in-hospital arrests)*

Patient details *(or hospital label)*

Family name

First name

Postcode

NHS/CHI/H&C number

Case note number

Date of birth *(dd/mm/yyyy)* / /

History at admission

FOR OUT-OF-HOSPITAL CARDIAC ARREST ONLY:
Bystander CPR attempted?

☐ Yes ☐ No ☐ Unknown

Did CPR continue after arrival to the Emergency Department?

☐ Yes ☐ No ☐ Unknown

FOR IN AND OUT-OF-HOSPITAL CARDIAC ARREST:

First monitored cardiac rhythm during cardiac arrest

- ☐ Asystole
- ☐ Sinus bradycardia < 60 bpm
- ☐ Pulseless electrical activity
- ☐ Ventricular fibrillation
- ☐ Ventricular tachycardia

if rhythm detected by ECG

- ☐ Shockable
- ☐ Non-shockable

if rhythm detected by automated external defibrillator (AED)

- ☐ No monitoring
- ☐ Unknown

Time from observed cardiac arrest to start of sustained return of spontaneous circulation (ROSC)

hours minutes

Number of doses of epinephrine from initial resuscitation to start of period of sustained ROSC

Temperature management

Core body temperature management during first 24 hours after sustained ROSC

- ☐ Active Normothermia *(35 to 37.9 °C)*
- ☐ Active Therapeutic Hypothermia *(32 to <35 °C)*
- ☐ Other *(state below)*
- ☐ No active temperature control
- ☐ Unknown

Duration of initial active temperature control management *(if temperature actively managed)*

hours

Minimum temperature recorded during first 24 hours

. °C

Maximum temperature recorded during first 24 hours

. °C

Comments

Form completed by

Contact us: picanet@leeds.ac.uk

Jodie Batchelor/Sophie Butler<br>*Project officer*<br>(0113) 343 8125<br>j.a.batchelor@leeds.ac.uk/s.butler1@leeds.ac.uk	Lee Norman<br>*Database manager*<br>(0113) 343 8125<br>l.j.norman@leeds.ac.uk	Caroline Lamming<br>*Research nurse*<br>(0116) 252 5414<br>crl4@leicester.ac.uk

www.picanet.org.uk PICANet custom audit data collection form · NET-PACK 3 · Version 1.3 · April 2017 · Copyright © 2017 Universities of Leeds and Leicester

2. When the PICU enters or uploads to PICANet Web the admission event data for the patient, completion of the PIM field **Cardiac arrest before ICU admission** will permit manual entry of NET-PACK 3 data items.

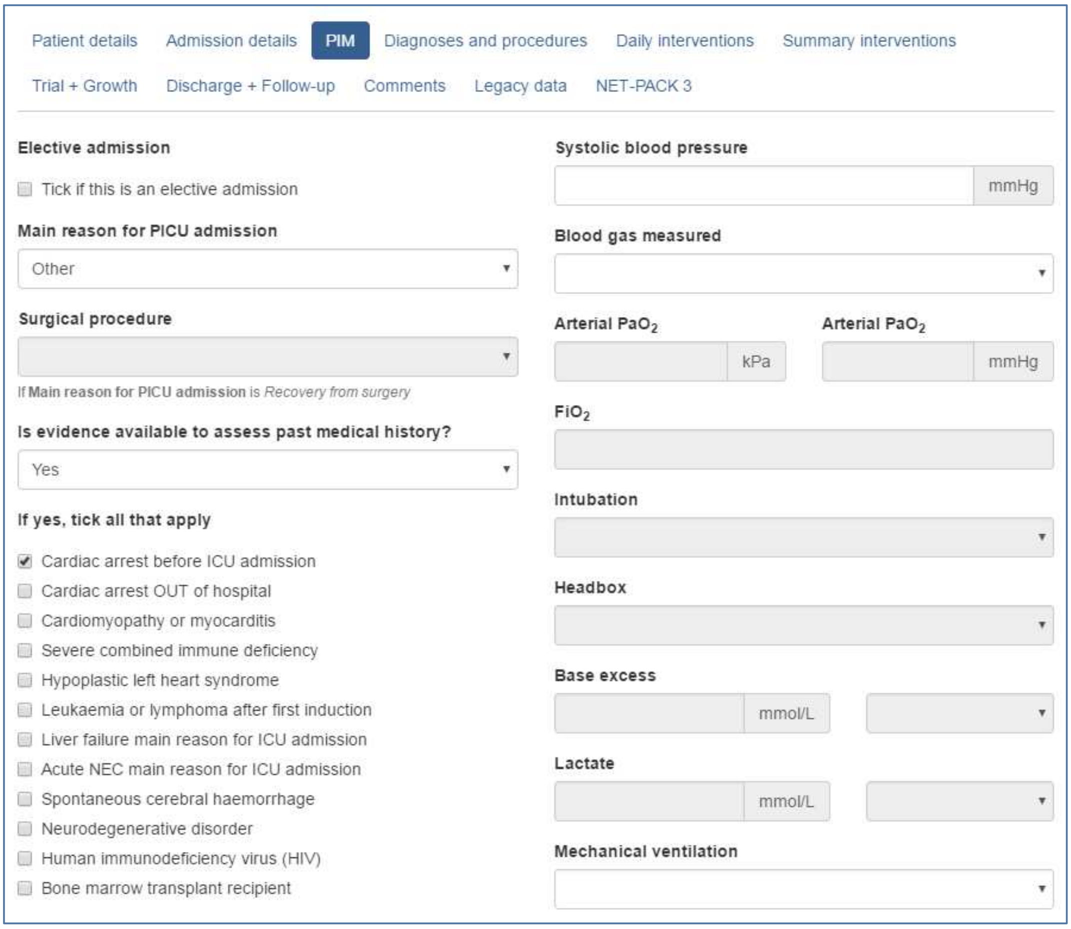

3. To enter NET-PACK 3 data, click the NET-PACK 3 tab. Note that the NET-PACK 3 tab is only visible for applicable events, i.e. when **Cardiac arrest before ICU admission** is ticked.

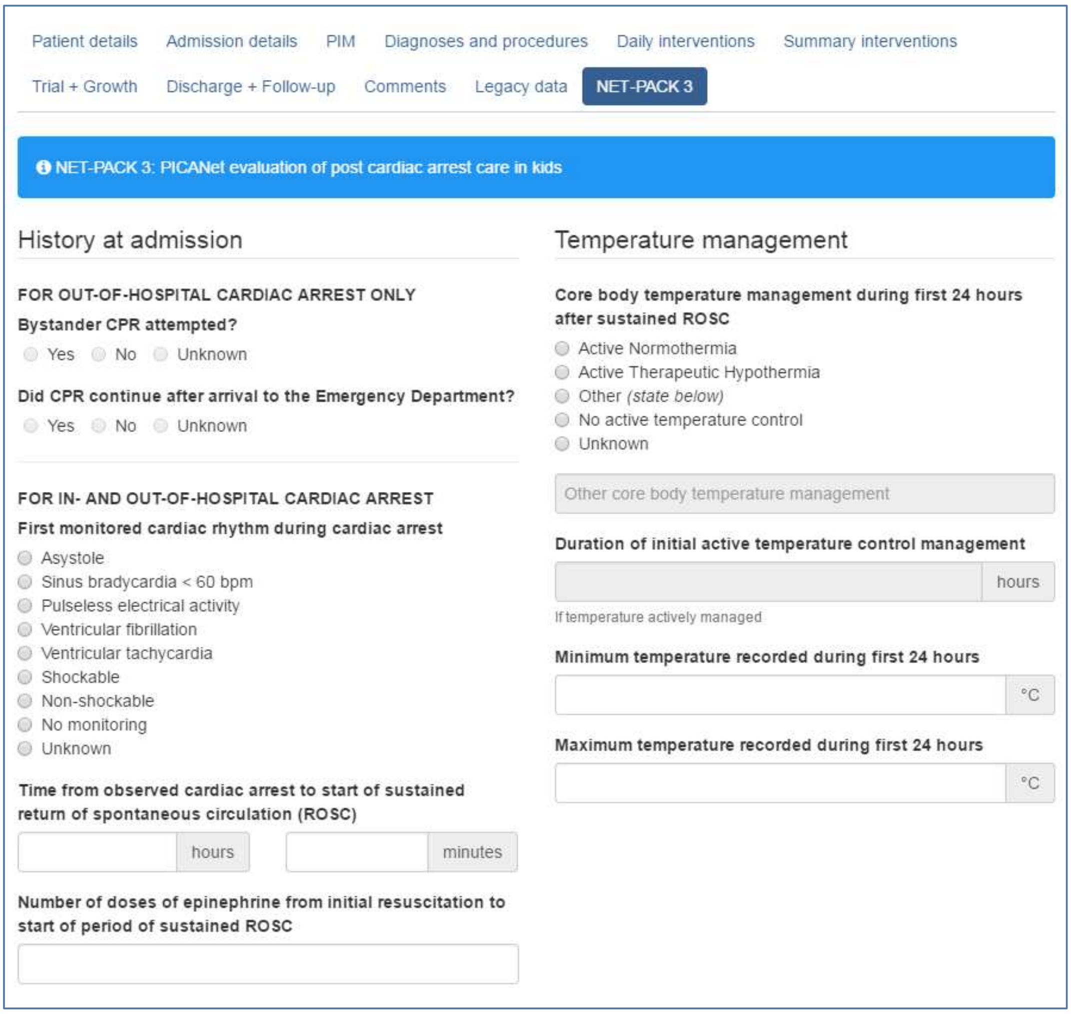

Scholefield BR, *et al. BMJ Open* 2020; 10:e037517. doi: 10.1136/bmjopen-2020-037517

Patient details

Family name or Surname

**Definition**	The last or family name or surname given to the child as it would appear on the child's birth certificate or other appropriate document.
**Reason**	Family name provides an additional identifier that can aid patient tracking throughout the hospital and PICANet Web.<br>Can help identify individuals who may have had multiple admissions to one or more PICUs.
**Format**	Free text (e.g. Brown).<br>If no family name available record as UNKNOWN and indicate why not available in the comments section.

First name

**Definition**	The first name given to the child as it would appear on the child's birth certificate or other appropriate document.
**Reason**	First name provides an additional identifier that can aid patient tracking throughout the hospital and PICANet Web.<br>Can help identify individuals who may have had multiple referrals and /or admissions to one or more PICUs.
**Format**	Free text (e.g. John).<br>If no first name available record as UNKNOWN and indicate why not available in the comments section.

Postcode

**Definition**	The postcode for the child's normal place of residence.
**Reason**	Postcode provides an additional identifier that can aid patient tracking throughout the hospital and PICANet Web.<br>Can help identify individuals who may have had multiple admissions to one or more PICUs.<br>Postcode provides a means of linkage to geographic and demographic information for effective audit and assessment of health services delivery.
**Format**	Text (e.g. S10 8NN).<br>Foreign postcodes will be accepted by the software, although a warning will be generated in the case of non UK standard postcodes to ensure that the user checks the data.<br>If postcode is unobtainable, record as UNKNOWN

NHS, CHI or H&C number

**Definition**	Unique identifying number enabling tracing of a patient through the NHS system in England, Wales and Northern Ireland. For English and Welsh patients the NHS number, for Scottish patients the CHI number and for Northern Ireland the H&C number is used as a unique numeric identifier.
**Reason**	NHS, CHI or H&C number gives a unique, identifiable variable that will allow other identifiable data items to be removed from the database.<br><br>Can help identify individuals who may have had multiple referrals, transport and/or admission events to one or more PICUs.
**Format**	Free text (e.g. 1463788990).<br>Validation check that NHS, CHI or H&C number is a valid number

Case note number

**Definition**	Unique identifying number for an individual's hospital records at the treating unit.<br>Allocated on first admission to hospital.
**Reason**	Case note number provides a unique identifier that can aid patient tracking throughout the hospital.
**Format**	Free text (e.g. AB145C).

Date of birth

**Definition**	The child's date of birth as recorded on the child's birth certificate or other appropriate document.
**Reason**	Date of birth and Date of admission are used to calculate age at admission to your unit.<br>Date of birth provides an additional identifier that can aid patient tracking throughout the hospital and PICANet Web.<br>Can help identify individuals who may have had multiple referrals and/or admissions to one or more PICUs.
**Format**	Date; dd/mm/yyyy.<br>Date of birth should be on or prior to the Date of admission.<br>If the child's date of birth is unobtainable, but the child is under your care, use your judgement to estimate year of birth and record as 1 January of estimated year (e.g. 01/01/YYYY).<br>If information is being extracted from notes and the child's date of birth is not recorded, or recorded as unavailable, leave the field blank and in the 'Indicate if date of birth is' field below tick 'Unknown'.<br>If it is necessary for Date of birth to be partly anonymised, enter the correct month and year and record 01 for the day (e.g. 01/MM/YYYY). Then tick 'Anonymised' below.
**Validation rule**	Warning if patient is aged 18 years or older

History at admission

Bystander Cardiopulmonary Resuscitation (CPR) Attempted?

For Out-of-Hospital Cardiac Arrest Only

**Definition**	Bystander cardiopulmonary resuscitation (CPR) is CPR performed by a person who is not responding as part of an organized emergency response system approach to a cardiac arrest. Physicians, nurses, and paramedics may be described as performing bystander CPR if they are not part of the emergency response system involved in the victim's resuscitation
**Reason**	Recording of this clinical variable can be used to validate a prediction model for hospital survival after out of hospital cardiac arrest.
**Format**	Yes<br>No<br>Unknown
**Validation rule**	Warning if value not entered

Cardiopulmonary Resuscitation continued after arrival to the Emergency Department?

For Out-of-Hospital Cardiac Arrest Only

**Definition**	If cardiac arrest and on-going cardiopulmonary resuscitation started in the pre-hospital setting AND continued after arrival in the emergency department record please indicate.
**Reason**	Failure to achieve a return of spontaneous circulation (ROSC) in the pre-hospital setting for out of hospital cardiac arrest patients is an important prognostic variable.
**Format**	Yes<br>No<br>Unknown
**Validation rule**	Warning if value not entered

Scholefield BR, *et al. BMJ Open* 2020; 10:e037517. doi: 10.1136/bmjopen-2020-037517

First monitored cardiac rhythm during cardiac arrest

**Definition**	Specifies the first cardiac rhythm present when a monitor or defibrillator is attached to a patient during a cardiac arrest.<br>If the automated external defibrillator (AED) does not have a rhythm display, then it may be possible to determine the first monitored rhythm from a data storage card, hard drive, or other device used by the AED to record data.<br>If initial rhythm is detected by an automated electrical defibrillator (AED) with no recording device, record whether the cardiac rhythm was shockable or non-shockable. If there is no ECG monitoring during cardiac arrest, record no monitoring.
**Reason**	Recording of this clinical variable can be used to validate a prediction model for hospital survival after out of hospital cardiac arrest.
**Format**	If rhythm detected by ECG choose from :<br>Asystole<br>Sinus bradycardia (defined < 60 beats per minute).<br>Pulseless electrical activity,<br>Ventricular fibrillation,<br>Ventricular tachycardia<br>if rhythm detected by an AED without an ECG readout use options:<br>Shockable,<br>Non-shockable<br>if no monitoring during cardiac arrest record<br>No monitoring<br>Unknown
**Validation rule**	Warning if value not entered

Time from observed cardiac arrest to start of sustained return of spontaneous circulation (ROSC)

**Definition**	Time from observed cardiac arrest to start of sustained return of spontaneous circulation (sustained ROSC*) The start time of the cardiac arrest will be the time reported when the child is first identified (found) in cardiac arrest by any bystander e.g. family, public, medical first responder. Estimation of period of time prior to this, which is unwitnessed, will not be included in the duration of cardiac arrest calculation.<br>Sustained Return of Spontaneous Circulation (Sustained ROSC) is deemed to have occurred when chest compressions are not required for 20 consecutive minutes and signs of circulation persist (or Return of circulation by extracorporeal circulatory support, if applied). The ‘start’ time will be when the initial ROSC (successful resuscitation and the restoration of a spontaneous perfusing rhythm) occurs except where patient has a further cardiac arrest within 20 mins of ROSC. The use of the start time of period of sustained ROSC will therefore take into account multiple cardiac arrests in the initial resuscitation period.

Scholefield BR, *et al. BMJ Open* 2020; 10:e037517. doi: 10.1136/bmjopen-2020-037517

**Reason**	Duration of cardiac arrest is required to calculate a prediction model for hospital survival after out of hospital cardiac arrest.
**Format**	Total number of hours and minutes<br>[_] hours [__] minutes
**Expected range**	0:01-8:00hrs
**Validation rule**	Validation check if time exceeds 8hrs: 00mins<br>Warning if value not entered

Number of doses of epinephrine from initial resuscitation to start of period of sustained ROSC

**Definition**	Record the total number of individual dose(s) of epinephrine (adrenaline), administered (via any route) from the commencement of initial resuscitation to the start of a period of sustained return of spontaneous circulation greater than 20 minutes (sustained ROSC).
**Reason**	An 'Utstein' defined variable required to calculate a prediction model for hospital survival after out of hospital cardiac arrest.
**Format**	Numerical value e.g.06
**Expected range**	00 – 40 validation check if number exceeds 40<br>99 if unknown
**Validation rule**	Validation check if number exceeds 40<br>Warning if value not entered

Temperature management

Core body temperature management planned during first 24 hours after sustained ROSC

**Definition**	The mode of core body temperature management during the first 24 hours after sustained return of spontaneous circulation (sustained ROSC)<br>**Active Normothermia** - defined as the active maintenance of core body temperature between 35 and <38 degrees Celsius)<br>**Active Therapeutic Hypothermia** - defined as active reduction of core body temperature to between 32 to <35 degrees Celsius)<br>**Other -** (complete comments box)<br>**No active temperature control**<br>**Unknown**
**Reason**	An 'Utstein' defined variable required to calculate a prediction model for hospital survival after out of hospital cardiac arrest.

Scholefield BR, *et al. BMJ Open* 2020; 10:e037517. doi: 10.1136/bmjopen-2020-037517

**Format**	Choose from one of the following:<br>Active Normothermia<br>Active Therapeutic hypothermia -<br>Other - complete text box<br>No active temperature control<br>Unknown
**Validation rule**	Warning if value not entered

Duration of initial active temperature control management

**Definition**	The duration of active temperature management if the core body temperature is actively managed by normothermia, therapeutic hypothermia or other stated method.
**Reason**	Required to provide further detail about active core body temperature processes
**Format**	Insert the total number of hours e.g.24 hours<br>if unknown insert 999
**Expected range**	1 – 120 hrs.
**Validation rule**	Validation check if number exceeds 120<br>Warning if temperature management type = Normothermia, Therapeutic hypothermia or other and no value added

Minimum temperature recorded during first 24 hours

**Definition**	The minimum temperature recorded during the first 24 hours after start of sustained return of spontaneous circulation (sustained ROSC).
**Reason**	Required to provide further detail about active core body temperature processes.
**Format**	Record in degrees Celsius e.g. 32.5 °C<br>if unknown record 999
**Expected range**	20.00-42 00 °C
**Validation rule**	Validation check if number exceeds 42.00 °C<br>Add warning if value not entered

Maximum temperature recorded during first 24 hours

**Definition**	The maximum temperature recorded during the first 24hours after start of sustained return of spontaneous circulation (sustained ROSC).
**Reason**	Required to provide further detail about active core body temperature processes.

Scholefield BR, *et al. BMJ Open* 2020; 10:e037517. doi: 10.1136/bmjopen-2020-037517

**Format**	Record in degrees Celsius e.g. 37.5°C<br>if unknown record 999
**Expected range**	20.00-42.00°C
**Validation rule**	Validation check if number exceeds 42.00 °C<br>Add warning if value not entered<br>Add warning if maximum temperature <= minimum temperature

Comments

**Definition**	Any additional information considered relevant to the dataset.<br>Text entered in this field may provide extra information about data entered elsewhere in a specific field in the dataset, or may provide extra information on the admission, which is not collected as part of the dataset.<br>No identifiers (patient, nurse, doctor, ICU, hospital) should be included in text data entered into this field.<br>As there is limited space in this field all text data should be kept to a minimum and be as concise as possible. Text data must not contain any punctuation except a period (full-stop) at the end of each data point.
**Reason**	No dataset specification covers all eventualities: to deal with this a text field has been included for comments/additional information.
**Format**	Free text

Form completed by

**Definition**	Name of person completing form.
**Reason**	For local use only to assist with following up queries relating to completion of this form.
**Format**	Free text
