# Supplementary material for: NEUROlogical Prognosis After Cardiac Arrest in Kids (NEUROPACK) study: protocol for a prospective multicentre clinical prediction model derivation and validation study in children after cardiac arrest
Source: BMJ Open. 2020 Sep 25;10(9):e037517. doi: 10.1136/bmjopen-2020-037517 (PMC7520830; doi:10.1136/bmjopen-2020-037517)
Supplement: Supplementary data [file bmjopen-2020-037517supp002.pdf]

Paediatric Intensive Care Audit Network · Custom Audit

NET-PACK 3

NET-PACK 3: PICANet evaluation of post cardiac arrest care in kids

Please complete for ***all PIC admissions following cardiac arrest*** *(include both out-of-hospital and in-hospital arrests)*

Patient details *(or hospital label)*

Family name

First name

Postcode

NHS/CHI/H&C number

Case note number

Date of birth *(dd/mm/yyyy)*

/ /

History at admission

FOR OUT-OF-HOSPITAL CARDIAC ARREST ONLY:
Bystander CPR attempted?

☐ Yes ☐ No ☐ Unknown

Did CPR continue after arrival to the Emergency Department?

☐ Yes ☐ No ☐ Unknown

FOR IN AND OUT-OF-HOSPITAL CARDIAC ARREST:

First monitored cardiac rhythm during cardiac arrest

- ☐ Asystole
- ☐ Sinus bradycardia < 60 bpm
- ☐ Pulseless electrical activity
- ☐ Ventricular fibrillation
- ☐ Ventricular tachycardia

if rhythm detected by ECG

- ☐ Shockable
- ☐ Non-shockable

if rhythm detected by automated external defibrillator (AED)

- ☐ No monitoring
- ☐ Unknown

Time from observed cardiac arrest to start of sustained return of spontaneous circulation (ROSC)

hours minutes

Number of doses of epinephrine from initial resuscitation to start of period of sustained ROSC

Temperature management

Core body temperature management during first 24 hours after sustained ROSC

- ☐ Active Normothermia *(35 to 37.9 °C)*
- ☐ Active Therapeutic Hypothermia *(32 to <35 °C)*
- ☐ Other *(state below)*
- ☐ No active temperature control
- ☐ Unknown

Duration of initial active temperature control management *(if temperature actively managed)*

hours

Minimum temperature recorded during first 24 hours

. °C

Maximum temperature recorded during first 24 hours

. °C

Comments

Form completed by

Contact us: picanet@leeds.ac.uk

Sophie Butler
Project officer
(0113) 343 8125
s.butler1@leeds.ac.uk

Lee Norman
Database manager
(0113) 343 8125
l.j.norman@leeds.ac.uk

Caroline Lamming
Research nurse
(0116) 252 5414
crl4@leicester.ac.uk

PICANet custom audit data collection form · NET-PACK 3 · Version 1.4 June 2017 · Copyright © 2017 Universities of Leeds and Leicester

Scholefield BR, *et al. BMJ Open* 2020; 10:e037517. doi: 10.1136/bmjopen-2020-037517
