# Supplementary material for: NEUROlogical Prognosis After Cardiac Arrest in Kids (NEUROPACK) study: protocol for a prospective multicentre clinical prediction model derivation and validation study in children after cardiac arrest
Source: BMJ Open. 2020 Sep 25;10(9):e037517. doi: 10.1136/bmjopen-2020-037517 (PMC7520830; doi:10.1136/bmjopen-2020-037517)
Supplement: Author's manuscript [file bmjopen-2020-037517.draft_revisions.pdf]

BMJ Open is committed to open peer review. As part of this commitment we make the peer review history of every article we publish publicly available.

When an article is published we post the peer reviewers' comments and the authors' responses online. We also post the versions of the paper that were used during peer review. These are the versions that the peer review comments apply to.

The versions of the paper that follow are the versions that were submitted during the peer review process. They are not the versions of record or the final published versions. They should not be cited or distributed as the published version of this manuscript.

BMJ Open is an open access journal and the full, final, typeset and author-corrected version of record of the manuscript is available on our site with no access controls, subscription charges or pay-per-view fees (http://bmjopen.bmj.com).

If you have any questions on BMJ Open's open peer review process please email info.bmjopen@bmj.com

BMJ Open

# NEUROlogical Prognosis After Cardiac Arrest in Kids (NEUROPACK) study: protocol for a prospective multicentre clinical prediction model derivation and validation study in children after cardiac arrest.



| | |
|---|---|
| Journal: | *BMJ Open* |
| Manuscript ID | bmjopen-2020-037517 |
| Article Type: | Protocol |
| Date Submitted by the Author: | 05-Feb-2020 |





| | |
|---|---|
| Complete List of Authors: | Scholefield, Barnaby; University of Birmingham College of Medical and Dental Sciences, Birmingham Acute Care Research Group; Birmingham Women and Children's NHS Foundation Trust, Paediatric Intensive Care Unit<br>Martin, James; University of Birmingham, Institute of Applied Health Research<br>Penny-Thomas, Kate; Birmingham Women and Children's NHS Foundation Trust, Paediatric Intensive Care Unit<br>Evans, Sarah; Birmingham Women and Children's NHS Foundation Trust, Paediatric Intensive Care Unit<br>Kool, Mirjam; University of Birmingham College of Medical and Dental Sciences, Birmingham Acute Care Research Group; Birmingham Women and Children's NHS Foundation Trust, Paediatric Intensive Care Unit<br>Parslow, Roger; University of Leeds, Leeds Institute for Data Analytics<br>Feltbower, Richard; University of Leeds, Leeds Institute for Data Analytics<br>Draper, Elizabeth; University of Leicester College of Medicine Biological Sciences and Psychology, Health Sciences<br>Hiley, Victoria; University of Leeds, Leeds Institute for Data Analytics<br>Sitch, Alice; University of Birmingham, Institute of Applied Health Research; University of Birmingham, NIHR Birmingham Biomedical Research Centre<br>Kanthimathinathan, Hari; Birmingham Women and Children's NHS Foundation Trust, Paediatric Intensive Care Unit ; University of Birmingham College of Medical and Dental Sciences, Birmingham Acute Care Research Group<br>Morris, Kevin; Birmingham Women's and Children's Hospital, Paediatric Intensive Care Unit; University of Birmingham, Institute of Applied Health Research<br>Smith, Fang; University of Birmingham College of Medical and Dental Sciences, Birmingham Acute Care Research Group |





| | |
|---|---|
| Keywords: | Paediatric intensive & critical care < INTENSIVE & CRITICAL CARE, Paediatric intensive & critical care < PAEDIATRICS, Protocols & guidelines < HEALTH SERVICES ADMINISTRATION & MANAGEMENT |
| | |

For peer review only - http://bmjopen.bmj.com/site/about/guidelines.xhtml

*I, the Submitting Author has the right to grant and does grant on behalf of all authors of the Work (as defined in the below author licence), an exclusive licence and/or a non-exclusive licence for contributions from authors who are: i) UK Crown employees; ii) where BMJ has agreed a CC-BY licence shall apply, and/or iii) in accordance with the terms applicable for US Federal Government officers or employees acting as part of their official duties; on a worldwide, perpetual, irrevocable, royalty-free basis to BMJ Publishing Group Ltd ("BMJ") its licensees and where the relevant Journal is co-owned by BMJ to the co-owners of the Journal, to publish the Work in this journal and any other BMJ products and to exploit all rights, as set out in our licence.*

*The Submitting Author accepts and understands that any supply made under these terms is made by BMJ to the Submitting Author unless you are acting as an employee on behalf of your employer or a postgraduate student of an affiliated institution which is paying any applicable article publishing charge ("APC") for Open Access articles. Where the Submitting Author wishes to make the Work available on an Open Access basis (and intends to pay the relevant APC), the terms of reuse of such Open Access shall be governed by a Creative Commons licence – details of these licences and which Creative Commons licence will apply to this Work are set out in our licence referred to above.*

*Other than as permitted in any relevant BMJ Author's Self Archiving Policies, I confirm this Work has not been accepted for publication elsewhere, is not being considered for publication elsewhere and does not duplicate material already published. I confirm all authors consent to publication of this Work and authorise the granting of this licence.*

For peer review only

**Protocol**

# NEUROlogical Prognosis After Cardiac Arrest in Kids (NEUROPACK) study: protocol for a prospective multicentre clinical prediction model derivation and validation study in children after cardiac arrest.


**Authors:**

Barnaby R Scholefield[1,2], James T Martin[3], Kate Penny-Thomas[2], Sarah Evans[2], Mirjam Kool[1,2], Roger Parslow[4], Richard Feltbower[4], Elizabeth S Draper[5], Victoria Hiley[4], Alice J Sitch[3,6], Hari Krishnan Kanthimathinathan[2,3], Kevin P Morris[2,3], Fang Gao Smith[1] On behalf of the NEUROPACK Investigators for the Paediatric Intensive Care Society-Study Group (PICS-SG)

[1] Birmingham Acute Care Research Group, Institute of Inflammation and Ageing, University of Birmingham, UK
[2] Paediatric Intensive Care Unit, Birmingham Women and Children's Hospital NHS Trust
[3] Institute of Applied Health Research, University of Birmingham, UK
[4] Leeds Institute for Data Analytics, School of Medicine, University of Leeds
[5] Department of Health Sciences, University of Leicester
6 NIHR Birmingham Biomedical Research Centre, University Hospitals Birmingham NHS Foundation Trust and University of Birmingham, UK

Corresponding author:
Dr Barnaby R Scholefield, Birmingham Acute Care Research Group, Institute of Inflammation and Ageing, University of Birmingham, UK
b.scholefield@bham.ac.uk


For peer review only



For peer review only - http://bmjopen.bmj.com/site/about/guidelines.xhtml

# Abstract:

**Introduction:**

Currently, we are unable to accurately predict mortality or neurological morbidity following resuscitation after paediatric out of hospital (OHCA) or in-hospital (IHCA) cardiac arrest. A clinical prediction model may improve communication with parents and families and risk stratification of patients for appropriate post-cardiac arrest care. This study aims to the derive and validate a clinical prediction model to predict, within one hour of admission to the paediatric intensive care unit (PICU), neuro-developmental outcome at three months after paediatric cardiac arrest.

**Methods and analysis:**

A prospective study of children (age: >24 hours and <16 years), admitted to one of the 27 participating PICUs in the UK and Ireland, following an OHCA or IHCA. Patients are included if requiring more than one minute of cardiopulmonary resuscitation and mechanical ventilation at PICU admission Children who had cardiac arrests in PICU or neonatal intensive care unit will be excluded. Candidate variables will be identified from data submitted to the Paediatric Intensive Care Audit Network (PICANet) registry. Primary outcome is neuro-developmental status, assessed at three months by telephone interview using the Vineland Adaptive Behavioural Score II questionnaire. A clinical prediction model will be derived using logistic regression with model performance and accuracy assessment. External validation will be performed using the Therapeutic Hypothermia After Pediatric Cardiac Arrest (THAPCA) trial dataset. We aim to identify 370 patients, with successful consent and follow up of 150 patients. Patient inclusion started 1st January 2018 and inclusion will continue over 18 months.

**Ethics and dissemination:**

Ethical review of this protocol was completed by 27th September 2017 at the Wales Research Ethics Committee 5, 17/WA/0306. The results of this study will be published in peer reviewed journals and presented in conferences.


**Trial Registration Number:** ClinicalTrials.gov NCT03574025

**Keywords:** Observational; Paediatric; Cardiac Arrest; PICU; Prognostication

**Word Count Abstract:** 294

**Word Count Main text**: 2835



For peer review only - http://bmjopen.bmj.com/site/about/guidelines.xhtml

# Article Summary

## Strengths and limitations of this study

- This study addresses the uncertainty around prognosis after cardiac arrest by deriving and validating a clinical prediction model.
- A clinical prediction model is likely to benefit not only clinicians, but also families, by improving communication around prognosis.
- We aim to increase understanding of neuro-developmental outcomes of children after cardiac arrest in the UK
- The low incidence and wide variety of causes of paediatric cardiac arrest are key challenges in prospective prognostic research in this population.

# Introduction

**Paediatric cardiac arrest**

Paediatric cardiac arrest (CA) is an uncommon but potentially catastrophic event for both children and their families. Cardiac arrest is defined as the cessation of cardiac mechanical activity occurring with absence of signs of circulation. Approximately 1500 infants or children per year suffer a cardiac arrest in the United Kingdom (UK) and Republic of Ireland (RoI) with between 250-350 admitted to a paediatric intensive care unit (PICU) for post resuscitation care.[1] Survival to PICU discharge for this population is achieved in 35-45% patients admitted to PICU after an OHCA and 45-55% after IHCA. However, 50% of survivors are estimated to have ongoing neuro-developmental disabilities despite advances in post-cardiac arrest management.[2 3] The high mortality and morbidity rates are often associated with the degree of brain injury from the hypoxic-ischaemic insult at the time of cardiac arrest.

**Prognostication after cardiac arrest**

Clinicians are currently unable to accurately predict survival with a good neuro-developmental outcome after cardiac arrest with any certainty due to a lack of data.[4-6] Clinicians can be pessimistic, optimistic or unnecessarily ambiguous in their predictions, and this affects the clarity of communication with families and the implementation of on-going treatment plans.[4] Improved prognostication is therefore a high priority for parents of children who have suffered a CA. In addition, early stratification of patients who may benefit from critical care interventions would also be a significant advancement in their treatment[7 8] and has been lacking in major studies to date.[2 3]

Several prognostic factors are associated with survival following paediatric CA, such as patient age and pre-existing co-morbidities[9], cardiac arrest characteristics (location, initial cardiac arrest rhythm,



For peer review only - http://bmjopen.bmj.com/site/about/guidelines.xhtml

duration of cardiac arrest, presence and actions of bystanders,[9 10] physiological observations (e.g. pupillary response, blood lactate, systolic blood pressure)[10-12] and specific medical interventions.[12 13] However, studies examining prognostic factors for good neuro-developmental outcome are much less frequent.

The importance and weighting of these factors in prognosis decision making is complex and in 2010 the International Liaison Committee On Resuscitation (ILCOR) consensus statement identified a significant gap in knowledge in prognostic modelling with children[5] with no additional 'high quality' data to inform the 2015 guidance.[14]

**Rationale for study**

Accurate early prediction of neuro-developmental outcomes may reduce uncertainty and improve communication with families. It may also provide better risk-stratification for clinical trials and individualised treatment of patients.  Furthermore, we aim to gain a better understanding of the epidemiology and neurodevelopmental outcomes of children after CA in the UK and RoI.

# Methods and analysis:

**Study Aims:**

The aim of the NEUROPACK study is to 1) derive a clinical prediction model using key factors prospectively collected from a cohort of patients, available within the first hour of PICU admission after paediatric cardiac arrest to predict good neuro-developmental outcome at three months, 2) externally validate the clinical prediction model using an existing paediatric cardiac arrest dataset and 3) describe the current epidemiology of cardiac arrest cases in the United Kingdom (UK) and Republic of Ireland (RoI).

**Study Design:**

This study is a multi-centre, nationwide, prospective observational study combining both registry and cohort data.

**Setting:**

Patients will be enrolled from 24 PICUs within the UK and RoI. All study sites admit infants and children following cardiac arrest and routinely submit audit data to the Paediatric Intensive Care Audit Network (PICANet) registry.

**Ongoing PICU Registry: PICANet and NET-PACK 3:**

Since 2002, PICANet has prospectively collected demographic, diagnostic, and interventional data along with PICU survival outcomes for patients admitted to PICUs in England and Wales and now collects data for patients across the United Kingdom and RoI.[15] This includes severity of illness variables to build the Paediatric Index of Mortality risk-adjustment models.[16]

PICANet is also conducting an ongoing customised data collection of post-cardiac arrest management: PICANet Post Arrest Care in Kids (NET-PACK 3) with data definition and data collection form (Supplemental material 1 & 2). NET-PACK 3 customised data collection includes resuscitation variables available within a few hours of the CA. Data are either collected within one hour of admission onto PICU or within one hour of the attendance at the patient's bedside of a specialist paediatric critical care team (e.g. a specialised retrieval team travels to another hospital without a



For peer review only - http://bmjopen.bmj.com/site/about/guidelines.xhtml

PICU). These variables include: 1) attempted bystander cardiopulmonary resuscitation (CPR), 2) duration of CPR, 3) requirement of CPR after arrival at emergency department, 4) number of doses of epinephrine (adrenaline) required and 5) initial presenting cardiac rhythm. These factors were chosen to comply with Utstein style CA reporting guidelines[17 18]. PICANet collects survival to PICU discharge outcome data for all admissions.

## Eligibility for NEUROPACK:

### Inclusion

All patients aged 24 hours up to 16th birthday admitted to PICU after OHCA or IHCA will be included. Cardiac arrest will be defined as requiring > 1minute CPR. Patients will be included if they require invasive (e.g. endotracheal) mechanical ventilation at PICU admission.

### Exclusion

Exclusion criteria include cardiac arrests occurring within a PICU or neonatal intensive care unit. For children who survive to PICU discharge we will exclude patients where the local clinical team at participating sites feel inclusion is inappropriate and/or parent/guardian or family member of children are unable to understand the telephone questionnaires for neuro-developmental outcome assessments in English. All patients under the age of 24 hours will be excluded due to potentially different aetiology of CA related to birth events.

#### Identification and screening

Patients for the NEUROPACK study will be identified via entry into the PICANet database and by local researchers at each site screening PICU admissions daily. 'Cardiac arrest preceding ICU admission – out of hospital or in-hospital' is a specific high risk category in the Paediatric Index of Mortality (PIM-3) risk-adjustment model and is recorded within one hour of PICU admission, or within one hour of the attendance at the patient's bedside of a specialist paediatric critical care team.[16]

### Recruitment for neuro-developmental outcome assessment

Parent/guardians of CA patients who are expected to survive to three months following CA will be approached by local research staff, trained in Good Clinical Practice, to consent for telephone questionnaire at three months post CA.

This is a very sensitive and difficult time for parents and guardians. The approach to parents or guardians of critical ill children for recruitment to the NEUROPACK study will therefore be handled sensitively. Local researchers will be trained to identify the appropriate time to consent, utilise passive information giving to reduce burden of information (e.g. Ethics committee-approved posters displayed in family rooms) and liaise with the medical team managing the patient to acknowledge ongoing clinical management issues. Local site investigator (or delegate) will re-contact parents or guardians at two months following CA to ascertain continued involvement in the study and to confirm ongoing contact details.

### Potential predictive factors collected

Potential candidate variables for the NEUROPACK clinical prediction model have been selected from the existing clinical prediction models for survival.[6 11 14] Final candidate variable selection will follow assessment of statistical modelling interaction and practicality of collecting variables in a timely fashion at the bedside by clinicians.

### Data collections



For peer review only - http://bmjopen.bmj.com/site/about/guidelines.xhtml

The ongoing NET-PACK 3 customised data collection and PICANet data collection for the PIM3 risk of mortality will be the data source for the candidate variables in the NEUROPACK study. Linkage of individual patient NET-PACK 3 data with the collected neuro-developmental outcome will be carried out for consented patients only. Pseudonymised data from NET-PACK 3 customised data collection and PICANET will be used for patients who die or for patients who survive and consent for follow up assessment is not available.

## Primary and secondary outcomes

### Primary outcome

The primary outcome is survival with a good neuro-developmental outcome at three months post event. Good neuro-developmental outcome is defined as a Vineland Adaptive Behaviour Scales 2$^{nd}$ edition (VABS-II) score of ≥ 70.[19]

### Primary outcome assessment

The VABS-II was designed as a caregiver report measure to assess communication, daily living, social, and motor domains of adaptive behaviour.[19] This tool can be used across the entire paediatric age range (0 to 16years) and requires a short interview which can be via telephone. VABS-II is sensitive to neurological injury and has been used successfully in paediatric neuro-critical care studies.[2] VABS-II has a normal mean value score of 100 (standard deviation of 15). Good neuro-developmental outcome is defined as a score of ≥ 70. Poor outcome is a composite score of VABS-II <70 and death. The chief investigator or the lead research nurse at the Central Research Centre (Birmingham Women & Children's NHS Foundation Trust, UK) will conduct all assessments. At the time of outcome assessment, the assessor will remain blinded to the clinical prediction model and component variables.

### Secondary outcomes

Paediatric cerebral performance category (PCPC) and paediatric overall performance category (POPC) at three months and change in PCPC and POPC score from baseline.[20] Survival to PICU discharge and three months post cardiac arrest.

### Secondary outcome assessment

PCPC and POPC scale can be calculated by a short questionnaire conducted at the 3 month follow up interview for consented patients. A baseline (pre-cardiac arrest) PCPC and POPC will also retrospectively ascertained at the three month follow up.  PCPC and POPC have been recommended for reporting in all paediatric CA studies. They score 1 to 6 (1: normal, 2: mild disability, 3: moderate disability, 4: severe disability, 5: vegetative state or coma and 6: death). They provide less detail but correlate reasonably well with VABS II.[21] This will allow comparison with other CA studies. Good neurodevelopmental outcome will be defined as PCPC score of 1 to 3 or no change from baseline. Poor outcome will be defined as a score of 4 or less, including death. Three months follow up time point is chosen following the International Liaison Committee On Resuscitation (ILCOR), core-outcome set for adults after cardiac arrest (COSCA) recommendation[22] and demonstration of minimal change between three and 12 month following cardiac arrest.[23]

## Statistical consideration

### Data analysis plan



For peer review only - http://bmjopen.bmj.com/site/about/guidelines.xhtml

The data will be manually reviewed for errors, missing data and outliers before analysis. Extreme values will be set to missing if they are deemed unlikely, based on their validity range. Descriptive analysis of the data will be reported. Continuous variables will be reported as either median and interquartile range (IQR) or mean and standard deviation (SD) based on the distribution. Categorical variables will be described in numbers, percentages or both.

**Table 1. Patient and cardiac arrest characteristics**

| **Table 1. Patient and cardiac arrest characteristics** |
|---|
| **Patient Demographic** |
| • **Age in years^** |
| • **Presence of PIM-3 'high risk' co-morbidities*[16]** |
| **Cardiac arrest characteristics and interventions:** |
| • **Location of cardiac arrest (IH & OHCA)*** <br> ***OHCA is assigned if chest compressions were initiated before hospital arrival*** |
| • **Aetiology of arrest (cardiac & non-cardiac)*** |
| • **Duration of cardiopulmonary resuscitation^** <br> • **Continuation of cardiopulomonary resuscitation after Emergency Department arrival (for OHCA only)*.** |
| • **Bystander cardiopulmonary resuscitation*** |
| • **Initial cardiac rhythm recorded during CA (shockable & non-shockable)*** |
| • **Doses of epinephrine (adrenaline) during cardiopulmonary resuscitation^** |
| • **Use of continuous vasoactive infusions within one hour of PICU admission*** |
| **Service characteristics:** |
| • **Requirement of inter-hospital transfer prior to PICU admission*** |
| • **Time of arrest day (07:00 – 18:59) or night (19:00 – 06:59)*** |
| **Physiological variables:** <br> ***Measured for PIM-3 calculation: within one hour of PICU admission or within one hour of the attendance at the patient's bedside of a specialist paediatric critical care team*** |
| • **Systolic blood pressure^** |
| • **Pupillary reaction to light (greater than 3mm and both fixed & other) *** |
| • **Blood lactate level^** |
| ***categorical data, ^continuous data.*** <br> ***PIM-3: Paediatric Index of Mortality 3 score, IH: in-hospital, OHCA: out-of-hospital cardiac arrest*** |

**Sample Size**

To reduce problematic bias and improve precision we aim for at least 10 events per variable considered for multivariable modelling[24]. Following pilot data collection, we calculate 250 CA patients per year are admitted to 27 UK and RoI PICUs, 125 (50%) will survive to PICU discharge and 70 (30%) per year will survive with good neuro-developmental outcome. To test 7 variables we estimate a requirement of 70 events (e.g. patients with good neuro-developmental outcome). 100% of non-survivors will be included (included in PICANet and NET-PACK 3 audit database). We anticipate 80% recruitment and consent rate of remaining survivors. We therefore require data collection over an 18-month period to recruit 370 patients. We anticipate that this would ensure successful consent and follow up of 150 patients, of whom 75 patients are estimated to have a good neuro-developmental outcome.

**Statistical methods for developing a prognostic model**



For peer review only - http://bmjopen.bmj.com/site/about/guidelines.xhtml

We will develop a prognostic model using logistic regression analysis of candidate variables and a good neuro-developmental outcome as the primary outcome variable. Multiple imputation (using chained equations) will be used for any variables with missing data considered in the model. Auxiliary variables will be used to aid the imputation. The number of imputed data sets used will be equal to the fraction of missing data[25].

Table 1 lists all candidate variables. Those variables deemed to be clinically important will be forced into the final model. Candidate variables will be retained if they benefit the model. The process will begin by fitting the full model and then performing backwards elimination, with a conservative significance level of 0.157.[26] For categorical variables, the category with the lowest *p*-value will dictate whether the variable is included in the final model.

All continuous variables will be left in their raw form to ensure no data was lost through dichotomisation or categorisation. It will be initially assumed that variables follow a linear trend, before fractional polynomials will be considered using the following powers: -2, -1, -0.5, natural logarithm, 0.5, 1, 2, and 3. A *p*-value <0.001 will be required to use a fractional polynomial rather than assuming a linear trend.[27] The use of fractional polynomials will also be considered for all continuous variables eliminated from the model to check whether this changes their inclusion status.

**Assessment of prognostic model performance**

Assessment of the fitted model will be achieved by estimating calibration and discrimination. A calibration plot will be produced by plotting the observed risk against the predicted risk and the calibration slope calculated. We expect the slope should be approximately 1 as the model developed will be developed using this data. To judge discrimination, the area under the receiver operating curve (equivalent to the c-statistic) and the R squared statistic will be calculated.

**Internal validation of the prognostic model**

The model will be internally validated using bootstrap methods. The original data will be used to generate 100 bootstrapped data sets. Each one of these bootstrapped data sets will then be used to develop a prognostic model in the same way as the original model. Estimates of performance (c-statistic and calibration slope) will be obtained from the model fitted using each of the bootstrapped data sets. The estimates obtained from the bootstrapped data sets will be averaged and subtracted from the estimates from the original model to estimate optimism and provide optimism-adjusted performance statistics.

**Final prognostic model**

The optimised adjusted calibration slope will then be used as a uniform shrinkage factor. Each of the coefficients from the original model will be adjusted for by multiplying by the shrinkage factor. The intercept will also be adjusted to ensure calibration-in-the-large, the average predicted probability, is the same as the average observed probability.

**Secondary analysis**

Using the secondary outcomes, we will repeat the steps above to create a supplemental final prognostic model, for survival to PICU and survival to three months. In addition we will create a prognostic model for good neurodevelopmental outcome using POPC and PCPC outcome scores.



For peer review only - http://bmjopen.bmj.com/site/about/guidelines.xhtml

**External Validation of the NEUROPACK prognostic score**

As part of the process of ensuring a prediction model is considered clinically useful, it must be validated in an external dataset.[28] We aim to do this by validating the NEUROPACK prognostic model in the publically accessible dataset for the Therapeutic Hypothermia After Pediatric Cardiac Arrest (THAPCA) OHCA and IHCA randomised controlled trials in the National Institute for Health Biolincc repository (Http://biolincc.nhlbi.nih.gov).[2 3] The sample size of the dataset to be used for external validation should be sufficient to provide reliable and accurate results. To externally validate the model, predictions of risk for each patient in the external validation dataset are made, and performance statistics, such as the C-statistic, are calculated in the same manner as described earlier.

# Patient and Public Involvement:

Given the sensitive and emotive nature of the NEUROPACK study, and the need for active parent and family engagement throughout, a patient advisory group, consisting of parents with experience of critical illness and death in children, and the Clinical Research Network: Children young person's advisory group (a sub group of the Generation R group aged 9-17yrs) have been consulted in designing the protocol, the informational material to support the intervention, and to understand the burden of the intervention from the patient's perspective. At the end of the study, the patient advisory group will be consulted on findings and contribute to the dissemination plan.

# Ethics and dissemination:

PICANet has ethical approval as a research database granted by the East Midlands, Derby Research Ethics Committee (ref 18/EM/0267) and NHS Health Research Authority Confidentiality Advisory Group approval (ref PIAG 4-07/(c)2002) to collect personally identifiable data without consent. The PICANet Clinical Advisory Group has approved pseudonymised sharing of PICANet audit data for the NEUROPACK study and Data Sharing Agreements will be established with the data controllers for the PICANet dataset prior to the release of de-identified PICANet and NET-PACK 3 data. Quality control of NET-PACK 3 customised data collection, data definitions and data collection is performed by the PICANet team.

Regional Ethics Committee (REC) permission has been obtained (Wales Research Ethics Committee 5, 17/WA/0306). This permits the ethical approach and consent of parents/guardians of eligible children who are likely to survive to 3 months following CA to enable telephone VABS-II assessment and identified data-linkage and sharing with PICANet and NET-PACK3 data.

We aim to publish the results in peer reviewed journals and present at relevant national and international conferences.

# Acknowledgements:

We would like to thank all participating sites for their contribution to data collection. We thank the NEUROPACK Principle Investigators from the Paediatric Intensive Care Society Study Group (PICS-SG) listed below:



For peer review only - http://bmjopen.bmj.com/site/about/guidelines.xhtml

1. Alder Hey Children's Hospital, Liverpool, UK. Dr Kent Thorburn
2. Birmingham Women and Children's Hospital NHS Foundation Trust, UK. Dr Barnaby Scholefield
3. Bristol Royal Hospital for Children, UK. Dr Rohit Saxena
4. Freeman Hospital, Newcastle, UK. Dr Yamuna Thiru
5. Glasgow Royal Hospital for Children, UK. Dr Richard Levin
6. Great North Children's Hospital, Newcastle, UK. Dr Rachel Agbecko
7. Great Ormond Street Hospital, London, UK. Dr Timothy Thiruchelvam & Dr Sophie Skellett
8. Imperial College Healthcare NHS Trust, London, UK. Dr David Inwald.
9. John Radcliff Hospital, Oxford, UK. Dr James Weitz
10. Kings College Hospital NHS Foundation Trust, London, UK. Dr Akash Deep
11. Leeds Children's Hospital, UK. Dr Sian Cooper
12. Leicester Children's Hospital, UK. Dr Peter Barry
13. Nottingham Children's Hospital, UK. Dr Patrick Davies
14. Our Ladies Children's Hospital, Crumlin, Dublin, Republic of Ireland. Dr Cormac Breatnach.
15. Royal Brompton Hospital, London, UK. Dr Sandra Gala-Peralta
16. Royal Hospital for Sick Children, Edinburgh, UK. Dr Milly Lo.
17. Royal Manchester Children's Hospital, UK. Dr Rachael Barber
18. Royal Victoria Hospital, Belfast, UK. Dr Stewart Reid.
19. Sheffield Children's NHS Foundation Trust, UK. Dr Rum Thomas.
20. Southampton General Hospital, UK. Dr John Pappachan.
21. St George's Hospital, London, UK. Dr Buvana Dwarakanathan.
22. The Noah's Ark Children's Hospital for Wales, UK. Cardiff. Dr Siva Oruganti.
23. The Royal London Hospital, UK. Dr Kalai Sadasivam.
24. University Hospitals of North Midlands, Stoke on Trent, UK. Dr Mark Bebbington

For peer review only



For peer review only - http://bmjopen.bmj.com/site/about/guidelines.xhtml

# References

1. Scholefield BR, Gao F, Duncan HP, et al. Observational study of children admitted to United Kingdom and Republic of Ireland Paediatric Intensive Care Units after out-of-hospital cardiac arrest. *Resuscitation* 2015 doi: 10.1016/j.resuscitation.2015.07.011 [published Online First: 2015/07/25]
2. Moler FW, Silverstein FS, Holubkov R, et al. Therapeutic Hypothermia after Out-of-Hospital Cardiac Arrest in Children. *The New England journal of medicine* 2015 doi: 10.1056/NEJMoa1411480 [published Online First: 2015/04/29]
3. Moler FW, Silverstein FS, Holubkov R, et al. Therapeutic Hypothermia after In-Hospital Cardiac Arrest in Children. *The New England journal of medicine* 2017;376(4):318-29. doi: 10.1056/NEJMoa1610493 [published Online First: 2017/01/25]
4. Fallowfield L, Jenkins V. Communicating sad, bad, and difficult news in medicine. *Lancet (London, England)* 2004;363(9405):312-9. doi: 10.1016/s0140-6736(03)15392-5 [published Online First: 2004/01/31]
5. Kleinman ME, de Caen AR, Chameides L, et al. Part 10: Pediatric basic and advanced life support: 2010 International Consensus on Cardiopulmonary Resuscitation and Emergency Cardiovascular Care Science With Treatment Recommendations. *Circulation* 2010;122(16 Suppl 2):S466-515. doi: 10.1161/CIRCULATIONAHA.110.971093 [published Online First: 2010/10/22]
6. Phillips RS, Scott B, Carter SJ, et al. Systematic Review and Meta-Analysis of Outcomes after Cardiopulmonary Arrest in Childhood. *PloS one* 2015;10(6):e0130327. doi: 10.1371/journal.pone.0130327 [published Online First: 2015/06/25]
7. Kent DM, Hayward RA. Limitations of applying summary results of clinical trials to individual patients: the need for risk stratification. *Jama* 2007;298(10):1209-12. doi: 10.1001/jama.298.10.1209 [published Online First: 2007/09/13]
8. Hingorani AD, Windt DA, Riley RD, et al. Prognosis research strategy (PROGRESS) 4: stratified medicine research. *BMJ (Clinical research ed)* 2013;346:e5793. doi: 10.1136/bmj.e5793 [published Online First: 2013/02/07]
9. Atkins DL, Everson-Stewart S, Sears GK, et al. Epidemiology and outcomes from out-of-hospital cardiac arrest in children: the Resuscitation Outcomes Consortium Epistry-Cardiac Arrest. *Circulation* 2009;119(11):1484-91.
10. Moler FW, Donaldson AE, Meert K, et al. Multicenter cohort study of out-of-hospital pediatric cardiac arrest. *Crit Care Med* 2011;39(1):141-9. doi: 10.1097/CCM.0b013e3181fa3c17 [published Online First: 2010/10/12]
11. Scholefield BR, Gao F, Duncan HP, et al. Observational study of children admitted to United Kingdom and Republic of Ireland Paediatric Intensive Care Units after out-of-hospital cardiac arrest. *Resuscitation* 2015;97:122-8. doi: 10.1016/j.resuscitation.2015.07.011 [published Online First: 2015/07/25]
12. Meert KL, Telford R, Holubkov R, et al. Pediatric Out-of-Hospital Cardiac Arrest Characteristics and Their Association With Survival and Neurobehavioral Outcome. *Pediatric critical care medicine : a journal of the Society of Critical Care Medicine and the World Federation of*



For peer review only - http://bmjopen.bmj.com/site/about/guidelines.xhtml

*Pediatric Intensive and Critical Care Societies* 2016;17(12):e543-e50. doi: 10.1097/pcc.0000000000000969 [published Online First: 2016/09/30]

13. Rodriguez-Nunez A, Lopez-Herce J, Garcia C, et al. Effectiveness and long-term outcome of cardiopulmonary resuscitation in paediatric intensive care units in Spain. *Resuscitation* 2006;71(3):301-9.
14. American Heart Association. Post ROSC predictive factors for survival in infants and children. 2015. https://volunteer.heart.org/apps/pico/Pages/PublicComment.aspx?q=813 (accessed 1st June 2015).
15. PICANET. The Paediatric Intensive Care Audit Network (PICANet) Project protocol. 2008. https://www.picanet.org.uk/wp-content/uploads/sites/25/2020/01/PICANet-project-protocol-v4.0_2018_08Aug_08.pdf (accessed 01 July 2019).
16. Straney L, Clements A, Parslow RC, et al. Paediatric index of mortality 3: an updated model for predicting mortality in pediatric intensive care*. *Pediatric critical care medicine : a journal of the Society of Critical Care Medicine and the World Federation of Pediatric Intensive and Critical Care Societies* 2013;14(7):673-81.
17. Perkins GD, Jacobs IG, Nadkarni VM, et al. Cardiac Arrest and Cardiopulmonary Resuscitation Outcome Reports: Update of the Utstein Resuscitation Registry Templates for Out-of-Hospital Cardiac Arrest: A Statement for Healthcare Professionals From a Task Force of the International Liaison Committee on Resuscitation (American Heart Association, European Resuscitation Council, Australian and New Zealand Council on Resuscitation, Heart and Stroke Foundation of Canada, InterAmerican Heart Foundation, Resuscitation Council of Southern Africa, Resuscitation Council of Asia); and the American Heart Association Emergency Cardiovascular Care Committee and the Council on Cardiopulmonary, Critical Care, Perioperative and Resuscitation. *Resuscitation* 2015;96:328-40. doi: 10.1016/j.resuscitation.2014.11.002 [published Online First: 2014/12/02]
18. Zaritsky A, Nadkarni V, Hazinski MF, et al. Recommended guidelines for uniform reporting of pediatric advanced life support: the pediatric Utstein style. A statement for healthcare professionals from a task force of the American Academy of Pediatrics, the American Heart Association, and the European Resuscitation Council. *Pediatrics* 1995;96(4 Pt 1):765-79. [published Online First: 1995/10/01]
19. Sparrow S, Cicchetti D, Balla D. Vineland Adaptive Behavior Scales (2nd edition). Circle Pines, MN: AGS Publishing 2005.
20. Fiser DH, Long N, Roberson PK, et al. Relationship of pediatric overall performance category and pediatric cerebral performance category scores at pediatric intensive care unit discharge with outcome measures collected at hospital discharge and 1- and 6-month follow-up assessments. *Critical care medicine* 2000;28(7):2616-20.
21. Slomine BS, Silverstein FS, Christensen JR, et al. Neuropsychological Outcomes of Children 1 Year After Pediatric Cardiac Arrest: Secondary Analysis of 2 Randomized Clinical Trials. *JAMA Neurol* 2018;75(12):1502-10. doi: 10.1001/jamaneurol.2018.2628 [published Online First: 2018/09/23]
22. Haywood K, Whitehead L, Nadkarni VM, et al. COSCA (Core Outcome Set for Cardiac Arrest) in Adults: An Advisory Statement From the International Liaison Committee on Resuscitation. *Resuscitation* 2018;127:147-63. doi: 10.1016/j.resuscitation.2018.03.022 [published Online First: 2018/05/01]
23. Slomine BS, Silverstein FS, Christensen JR, et al. Neurobehavioural outcomes in children after In-Hospital cardiac arrest. *Resuscitation* 2018;124:80-89. doi: 10.1016/j.resuscitation.2018.01.002 [published Online First: 2018/01/07]
24. Peduzzi P, Concato J, Kemper E, et al. A simulation study of the number of events per variable in logistic regression analysis. *J Clin Epidemiol* 1996;49(12):1373-9. [published Online First: 1996/12/01]



For peer review only - http://bmjopen.bmj.com/site/about/guidelines.xhtml

25. White IR, Royston P, Wood AM. Multiple imputation using chained equations: Issues and guidance for practice. *Stat Med* 2011;30(4):377-99. doi: 10.1002/sim.4067 [published Online First: 2011/01/13]
26. Sauerbrei W. The Use of Resampling Methods to Simplify Regression Models in Medical Statistics. *Journal of the Royal Statistical Society Series C (Applied Statistics)* 1999;48(3):313-29.
27. Royston P, Altman DG. Regression Using Fractional Polynomials of Continuous Covariates: Parsimonious Parametric Modelling. *Journal of the Royal Statistical Society Series C (Applied Statistics)* 1994;43(3):429-67. doi: 10.2307/2986270
28. Collins GS, de Groot JA, Dutton S, et al. External validation of multivariable prediction models: a systematic review of methodological conduct and reporting. *BMC Med Res Methodol* 2014;14:40. doi: 10.1186/1471-2288-14-40 [published Online First: 2014/03/22]

For peer review only

## Authors' contributions

BS: initiated the collaborative project, designed the study, designed data collection tools, monitored data collection for the whole trial, wrote the statistical analysis plan, drafted and revised the paper. He is guarantor. JM: wrote the statistical analysis plan, revised the paper. KP-T designed data collection tools, monitored data collection for the whole trial, revised the paper. AS: wrote the statistical analysis plan, revised the paper. RP, RF, ESD and VH: advised on PICANet data utility, monitored data collection for PICANET and revised the paper. SE, MK, HKK, KM, FGS: designed the study, drafted and revised the paper.

## Funding statement

Dr Barnaby Scholefield is funded by a National Institute for Health Research (NIHR) Clinician Scientist Fellowship (NIHR-CS-2015-15-016) for this research project.

## Consent

All patients and families will provide written informed consent/assent and will have the ability to withdraw at any time without explanation.

## Disclaimer

This publication presents independent research funded by the National Institute for Health Research (NIHR). The views expressed are those of the author(s) and not necessarily those of the NHS, the NIHR or the Department of Health and Social Care

## Conflicts of interests statement

None declared.

## Supplemental Material.



For peer review only - http://bmjopen.bmj.com/site/about/guidelines.xhtml

## 1. NETPACK 3 data definitions

**https://www.picanet.org.uk/wp-content/uploads/sites/25/2018/05/NET-PACK-3-Data-Definitions-v1.1.pdf**

## 2. NETPACK 3 data collection form

**https://www.picanet.org.uk/wp-content/uploads/sites/25/2018/05/PICANet-form-custom-audit-NET-PACK-3-v1.4-June-2017.pdf**



For peer review only - http://bmjopen.bmj.com/site/about/guidelines.xhtml

# PICANet Custom Audit Definitions

# NET-PACK 3

Version 1.1 (May 2017)

For peer review only

# Contents


Introduction ..... 2

Background ..... 2

References ..... 2

Data collection method ..... 3

Patient details ..... 6

Family name or Surname ..... 6

First name ..... 6

Postcode ..... 6

NHS, CHI or H&C number ..... 7

Case note number ..... 7

Date of birth ..... 7

History at admission ..... 8

Bystander Cardiopulmonary Resuscitation (CPR) Attempted? ..... 8

Cardiopulmonary Resuscitation continued after arrival to the Emergency Department? ..... 8

First monitored cardiac rhythm during cardiac arrest ..... 9

Time from observed cardiac arrest to start of sustained return of spontaneous circulation (ROSC) 9

Number of doses of epinephrine from initial resuscitation to start of period of sustained ROSC ..... 10

Temperature management ..... 10

Core body temperature management planned during first 24 hours after sustained ROSC ..... 10

Duration of initial active temperature control management ..... 11

Minimum temperature recorded during first 24 hours ..... 11

Maximum temperature recorded during first 24 hours ..... 11

Comments ..... 12

Form completed by ..... 12




For peer review only - http://bmjopen.bmj.com/site/about/guidelines.xhtml

# Introduction

## Background

The NET-PACK 3 Custom Audit - PICANet evaluation of Post cardiac Arrest Care in Kids, is a re-audit of patient management after cardiac arrest in UK and Irish PICUs.

Between June 2014 and December 2015, in collaboration with Dr Barney Scholefield (Chief Investigator) at Birmingham Children's Hospital PICU and the Paediatric Intensive Care Society (PICS), PICANet performed the NET-PACK **2** custom audit in 29 UK and Irish PICUs.

Additional data was collected about post cardiac arrest management for either out-of-hospital or in-hospital cardiac arrest prior to PICU admission in 400 infants and children. Eight resuscitation variables available at the time of PICU admission and the early proposed post cardiac arrest temperature management plans were collected. The key findings will be published in detail shortly.

Importantly wide variation in PICU post-arrest management has been identified and also opportunities to stratify the cardiac arrest population for targeted treatments.

NET-PACK 3 has been designed to investigate the impact and compliance with the new International guidance and research data on post-arrest care as part of the PICANet clinical audit function. In December 2015 the International Liaison Committee on Resuscitation (ILCOR) published up-to-date guidance on Paediatric Advanced Life Support and post-cardiac arrest management (1). In addition two large randomised controlled trials of targeted temperature management after paediatric cardiac arrest have been published (2, 3). The primary objective of the NET-PACK 3 custom audit will be to assess whether targeted temperature management (TTM) is used, the dose of TTM (duration and temperature) following the ILCOR 2015 guidance and trial recent publications and the effect on survival outcome. In addition the NET-PACK 3 Custom Audit data will be available for linkage in centres participating in the NIHR funded NEUROdevelopmental Prognositic after Cardiac Arrest in Kids Trial (NEURO-PACK). This trial will be evaluating more detailed neuro-developmental outcomes of patients after paediatric cardiac arrest.

## References

1. de Caen AR, Maconochie IK, Aickin R, Atkins DL, Biarent D, Guerguerian AM, et al. Part 6: Pediatric Basic Life Support and Pediatric Advanced Life Support: 2015 International Consensus on Cardiopulmonary Resuscitation and Emergency Cardiovascular Care Science With Treatment Recommendations. Circulation. 2015;132(16 Suppl 1):S177-203. Epub 2015/10/17.
2. Moler FW, Silverstein FS, Holubkov R, Slomine BS, Christensen JR, Nadkarni VM, et al. Therapeutic Hypothermia after In-Hospital Cardiac Arrest in Children. N Engl J Med. 2017;376(4):318-29. Epub 2017/01/25.
3. Moler FW, Holubkov R, Dean JM. Therapeutic Hypothermia in Children. N Engl J Med. 2015;373(10):980. Epub 2015/09/04.



For peer review only - http://bmjopen.bmj.com/site/about/guidelines.xhtml

# Data collection method

For units who agree to participate in this custom audit PICANet will enable access to the specific custom audit data collection tab on the data entry page:-

1. A PICANet NET-PACK 3 custom audit form (see below) is completed for all admissions for either out-of-hospital or in-hospital cardiac arrest prior to PICU admission.

PICA Net

**Paediatric Intensive Care Audit Network · Custom Audit** **NET-PACK 3**

*NET-PACK 3: PICANet evaluation of post cardiac arrest care in kids*

*Please complete for* ***all PIC admissions following cardiac arrest*** *(include both out-of-hospital and in-hospital arrests)*

**Patient details** ***(or hospital label)***

Family name ☐

First name ☐

Postcode ☐☐☐☐ ☐☐☐

NHS/CHI/H&C number ☐☐☐ ☐☐☐ ☐☐☐☐

Case note number ☐☐☐☐☐☐☐☐☐☐

Date of birth *(dd/mm/yyyy)* ☐☐ / ☐☐ / ☐☐☐☐

**History at admission**

***FOR OUT-OF-HOSPITAL CARDIAC ARREST ONLY:***

**Bystander CPR attempted?**

☐ Yes ☐ No ☐ Unknown

**Did CPR continue after arrival to the Emergency Department?**

☐ Yes ☐ No ☐ Unknown

***FOR IN AND OUT-OF-HOSPITAL CARDIAC ARREST:***

**First monitored cardiac rhythm during cardiac arrest**

☐ Asystole
☐ Sinus bradycardia < 60 bpm
☐ Pulseless electrical activity
☐ Ventricular fibrillation
☐ Ventricular tachycardia
} *if rhythm detected by ECG*

☐ Shockable
☐ Non-shockable
} *if rhythm detected by automated external defibrillator (AED)*

☐ No monitoring

☐ Unknown

**Time from observed cardiac arrest to start of sustained return of spontaneous circulation (ROSC)**

☐ hours ☐☐ minutes

**Number of doses of epinephrine from initial resuscitation to start of period of sustained ROSC**

☐☐

**Temperature management**

**Core body temperature management during first 24 hours after sustained ROSC**

☐ Active Normothermia *(35 to 37.9 °C)*
☐ Active Therapeutic Hypothermia *(32 to <35 °C)*
☐ Other *(state below)*
☐ No active temperature control
☐ Unknown

☐

**Duration of initial active temperature control management** *(if temperature actively managed)*

☐☐ hours

**Minimum temperature recorded during first 24 hours**

☐☐ . ☐ °C

**Maximum temperature recorded during first 24 hours**

☐☐ . ☐ °C

**Comments**

☐

**Form completed by**

☐

**Contact us: picanet@leeds.ac.uk**

Jodie Batchelor/Sophie Butler
*Project officer*
(0113) 343 8125
j.a.batchelor@leeds.ac.uk/s.butler1@leeds.ac.uk

Lee Norman
*Database manager*
(0113) 343 8125
l.j.norman@leeds.ac.uk

Caroline Lamming
*Research nurse*
(0116) 252 5414
crl4@leicester.ac.uk

www.picanet.org.uk PICANet custom audit data collection form · NET-PACK 3 · Version 1.3 · April 2017 · Copyright © 2017 Universities of Leeds and Leicester

2. When the PICU enters or uploads to PICANet Web the admission event data for the patient, completion of the PIM field **Cardiac arrest before ICU admission** will permit manual entry of NET-PACK 3 data items.

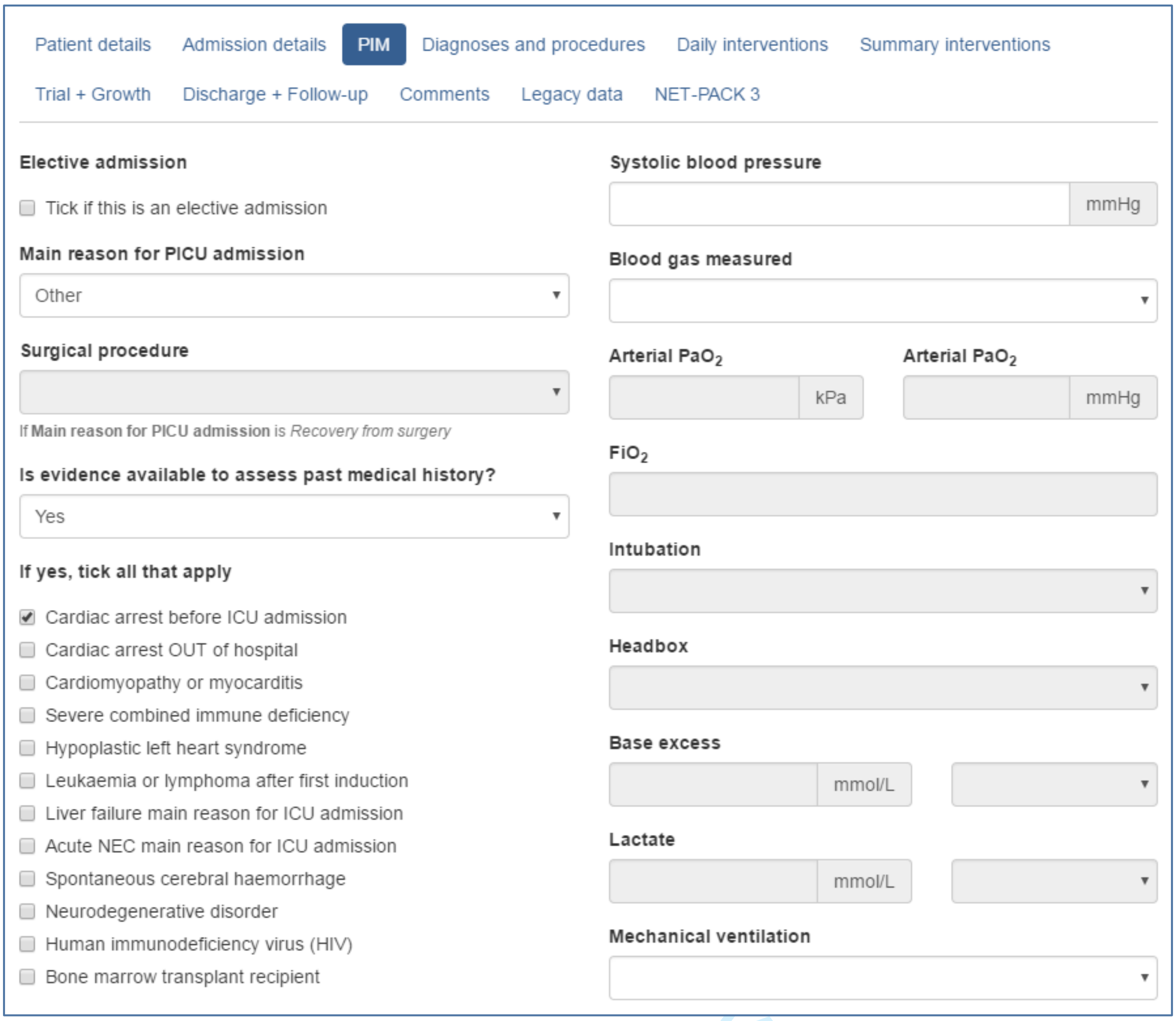




For peer review only - http://bmjopen.bmj.com/site/about/guidelines.xhtml

3. To enter NET-PACK 3 data, click the NET-PACK 3 tab. Note that the NET-PACK 3 tab is only visible for applicable events, i.e. when **Cardiac arrest before ICU admission** is ticked.

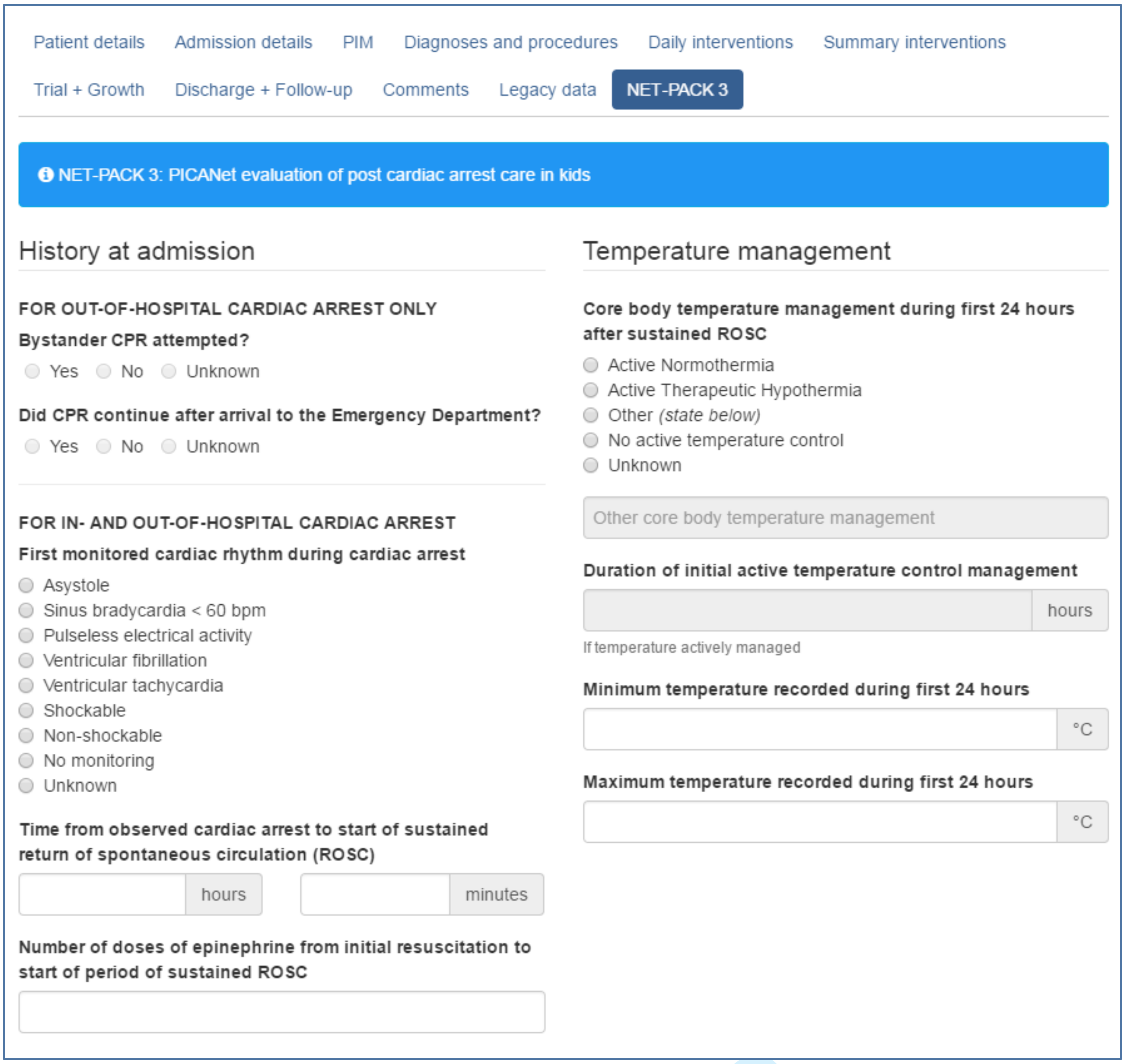
Patient details | Admission details | PIM | Diagnoses and procedures | Daily interventions | Summary interventions
Trial + Growth | Discharge + Follow-up | Comments | Legacy data | NET-PACK 3

NET-PACK 3: PICANet evaluation of post cardiac arrest care in kids

History at admission

FOR OUT-OF-HOSPITAL CARDIAC ARREST ONLY

Bystander CPR attempted?

Yes No Unknown

Did CPR continue after arrival to the Emergency Department?

Yes No Unknown

FOR IN- AND OUT-OF-HOSPITAL CARDIAC ARREST

First monitored cardiac rhythm during cardiac arrest

Asystole
Sinus bradycardia < 60 bpm
Pulseless electrical activity
Ventricular fibrillation
Ventricular tachycardia
Shockable
Non-shockable
No monitoring
Unknown

Time from observed cardiac arrest to start of sustained return of spontaneous circulation (ROSC)

hours minutes

Number of doses of epinephrine from initial resuscitation to start of period of sustained ROSC

Temperature management

Core body temperature management during first 24 hours after sustained ROSC

Active Normothermia
Active Therapeutic Hypothermia
Other *(state below)*
No active temperature control
Unknown

Other core body temperature management

Duration of initial active temperature control management

hours

If temperature actively managed

Minimum temperature recorded during first 24 hours

°C

Maximum temperature recorded during first 24 hours

°C



For peer review only - http://bmjopen.bmj.com/site/about/guidelines.xhtml

# Patient details

## Family name or Surname

| | |
|---|---|
| **Definition** | The last or family name or surname given to the child as it would appear on the child's birth certificate or other appropriate document. |
| **Reason** | Family name provides an additional identifier that can aid patient tracking throughout the hospital and PICANet Web.<br>Can help identify individuals who may have had multiple admissions to one or more PICUs. |
| **Format** | Free text (e.g. Brown).<br>If no family name available record as UNKNOWN and indicate why not available in the comments section. |

## First name

| | |
|---|---|
| **Definition** | The first name given to the child as it would appear on the child's birth certificate or other appropriate document. |
| **Reason** | First name provides an additional identifier that can aid patient tracking throughout the hospital and PICANet Web.<br>Can help identify individuals who may have had multiple referrals and /or admissions to one or more PICUs. |
| **Format** | Free text (e.g. John).<br>If no first name available record as UNKNOWN and indicate why not available in the comments section. |

## Postcode

| | |
|---|---|
| **Definition** | The postcode for the child's normal place of residence. |
| **Reason** | Postcode provides an additional identifier that can aid patient tracking throughout the hospital and PICANet Web.<br>Can help identify individuals who may have had multiple admissions to one or more PICUs.<br>Postcode provides a means of linkage to geographic and demographic information for effective audit and assessment of health services delivery. |
| **Format** | Text (e.g. S10 8NN).<br>Foreign postcodes will be accepted by the software, although a warning will be generated in the case of non UK standard postcodes to ensure that the user checks the data.<br>If postcode is unobtainable, record as UNKNOWN |



For peer review only - http://bmjopen.bmj.com/site/about/guidelines.xhtml

## NHS, CHI or H&C number

| | |
|---|---|
| **Definition** | Unique identifying number enabling tracing of a patient through the NHS system in England, Wales and Northern Ireland. For English and Welsh patients the NHS number, for Scottish patients the CHI number and for Northern Ireland the H&C number is used as a unique numeric identifier. |
| **Reason** | NHS, CHI or H&C number gives a unique, identifiable variable that will allow other identifiable data items to be removed from the database.<br><br>Can help identify individuals who may have had multiple referrals, transport and/or admission events to one or more PICUs. |
| **Format** | Free text (e.g. 1463788990).<br>Validation check that NHS, CHI or H&C number is a valid number |

## Case note number

| | |
|---|---|
| **Definition** | Unique identifying number for an individual's hospital records at the treating unit.<br>Allocated on first admission to hospital. |
| **Reason** | Case note number provides a unique identifier that can aid patient tracking throughout the hospital. |
| **Format** | Free text (e.g. AB145C). |

## Date of birth

| | |
|---|---|
| **Definition** | The child's date of birth as recorded on the child's birth certificate or other appropriate document. |
| **Reason** | Date of birth and Date of admission are used to calculate age at admission to your unit.<br>Date of birth provides an additional identifier that can aid patient tracking throughout the hospital and PICANet Web.<br>Can help identify individuals who may have had multiple referrals and/or admissions to one or more PICUs. |
| **Format** | Date; dd/mm/yyyy.<br>Date of birth should be on or prior to the Date of admission.<br>If the child's date of birth is unobtainable, but the child is under your care, use your judgement to estimate year of birth and record as 1 January of estimated year (e.g. 01/01/YYYY).<br>If information is being extracted from notes and the child's date of birth is not recorded, or recorded as unavailable, leave the field blank and in the 'Indicate if date of birth is' field below tick 'Unknown'.<br>If it is necessary for Date of birth to be partly anonymised, enter the correct month and year and record 01 for the day (e.g. 01/MM/YYYY). Then tick 'Anonymised' below. |
| **Validation rule** | Warning if patient is aged 18 years or older |



For peer review only - http://bmjopen.bmj.com/site/about/guidelines.xhtml

# History at admission

## Bystander Cardiopulmonary Resuscitation (CPR) Attempted?

### *For Out-of-Hospital Cardiac Arrest Only*

| | |
|---|---|
| **Definition** | Bystander cardiopulmonary resuscitation (CPR) is CPR performed by a person who is not responding as part of an organized emergency response system approach to a cardiac arrest. Physicians, nurses, and paramedics may be described as performing bystander CPR if they are not part of the emergency response system involved in the victim's resuscitation |
| **Reason** | Recording of this clinical variable can be used to validate a prediction model for hospital survival after out of hospital cardiac arrest. |
| **Format** | Yes<br>No<br>Unknown |
| **Validation rule** | Warning if value not entered |

## Cardiopulmonary Resuscitation continued after arrival to the Emergency Department?

### *For Out-of-Hospital Cardiac Arrest Only*

| | |
|---|---|
| **Definition** | If cardiac arrest and on-going cardiopulmonary resuscitation started in the pre-hospital setting AND continued after arrival in the emergency department record please indicate. |
| **Reason** | Failure to achieve a return of spontaneous circulation (ROSC) in the pre-hospital setting for out of hospital cardiac arrest patients is an important prognostic variable. |
| **Format** | Yes<br>No<br>Unknown |
| **Validation rule** | Warning if value not entered |

For peer review only

## First monitored cardiac rhythm during cardiac arrest

**Definition**

Specifies the first cardiac rhythm present when a monitor or defibrillator is attached to a patient during a cardiac arrest.

If the automated external defibrillator (AED) does not have a rhythm display, then it may be possible to determine the first monitored rhythm from a data storage card, hard drive, or other device used by the AED to record data.

If initial rhythm is detected by an automated electrical defibrillator (AED) with no recording device, record whether the cardiac rhythm was shockable or non-shockable. If there is no ECG monitoring during cardiac arrest, record no monitoring.

**Reason**

Recording of this clinical variable can be used to validate a prediction model for hospital survival after out of hospital cardiac arrest.

**Format**

If rhythm detected by ECG choose from :

- Asystole
- Sinus bradycardia (defined < 60 beats per minute).
- Pulseless electrical activity,
- Ventricular fibrillation,
- Ventricular tachycardia

if rhythm detected by an AED without an ECG readout use options:

- Shockable,
- Non-shockable

if no monitoring during cardiac arrest record

- No monitoring
- Unknown

**Validation rule**

Warning if value not entered

## Time from observed cardiac arrest to start of sustained return of spontaneous circulation (ROSC)

**Definition**

Time from observed cardiac arrest to start of sustained return of spontaneous circulation (sustained ROSC*) The start time of the cardiac arrest will be the time reported when the child is first identified (found) in cardiac arrest by any bystander e.g. family, public, medical first responder. Estimation of period of time prior to this, which is unwitnessed, will not be included in the duration of cardiac arrest calculation.

Sustained Return of Spontaneous Circulation (Sustained ROSC) is deemed to have occurred when chest compressions are not required for 20 consecutive minutes and signs of circulation persist (or Return of circulation by extracorporeal circulatory support, if applied). The 'start' time will be when the initial ROSC (successful resuscitation and the restoration of a spontaneous perfusing rhythm) occurs except where patient has a further cardiac arrest within 20 mins of ROSC. The use of the start time of period of sustained ROSC will therefore take into account multiple cardiac arrests in the initial resuscitation period.

For peer review only



For peer review only - http://bmjopen.bmj.com/site/about/guidelines.xhtml

| | |
|---|---|
| **Reason** | Duration of cardiac arrest is required to calculate a prediction model for hospital survival after out of hospital cardiac arrest. |
| **Format** | Total number of hours and minutes<br>[_] hours [__] minutes |
| **Expected range** | 0:01-8:00hrs |
| **Validation rule** | Validation check if time exceeds 8hrs: 00mins<br>Warning if value not entered |

## Number of doses of epinephrine from initial resuscitation to start of period of sustained ROSC

| | |
|---|---|
| **Definition** | Record the total number of individual dose(s) of epinephrine (adrenaline), administered (via any route) from the commencement of initial resuscitation to the start of a period of sustained return of spontaneous circulation greater than 20 minutes (sustained ROSC). |
| **Reason** | An 'Utstein' defined variable required to calculate a prediction model for hospital survival after out of hospital cardiac arrest. |
| **Format** | Numerical value e.g.06 |
| **Expected range** | 00 – 40 validation check if number exceeds 40<br>99 if unknown |
| **Validation rule** | Validation check if number exceeds 40<br>Warning if value not entered |

# Temperature management

## Core body temperature management planned during first 24 hours after sustained ROSC

| | |
|---|---|
| **Definition** | The mode of core body temperature management during the first 24 hours after sustained return of spontaneous circulation (sustained ROSC)<br>**Active Normothermia** - defined as the active maintenance of core body temperature between 35 and <38 degrees Celsius)<br>**Active Therapeutic Hypothermia** - defined as active reduction of core body temperature to between 32 to <35 degrees Celsius)<br>**Other -** (complete comments box)<br>**No active temperature control**<br>**Unknown** |
| **Reason** | An 'Utstein' defined variable required to calculate a prediction model for hospital survival after out of hospital cardiac arrest. |



For peer review only - http://bmjopen.bmj.com/site/about/guidelines.xhtml

**Format** Choose from one of the following:
- Active Normothermia
- Active Therapeutic hypothermia -
- Other - complete text box
- No active temperature control
- Unknown

**Validation rule** Warning if value not entered

## Duration of initial active temperature control management

**Definition** The duration of active temperature management if the core body temperature is actively managed by normothermia, therapeutic hypothermia or other stated method.

**Reason** Required to provide further detail about active core body temperature processes

**Format** Insert the total number of hours e.g.24 hours
if unknown insert 999

**Expected range** 1 – 120 hrs.

**Validation rule** Validation check if number exceeds 120
Warning if temperature management type = Normothermia, Therapeutic hypothermia or other and no value added

## Minimum temperature recorded during first 24 hours

**Definition** The minimum temperature recorded during the first 24 hours after start of sustained return of spontaneous circulation (sustained ROSC).

**Reason** Required to provide further detail about active core body temperature processes.

**Format** Record in degrees Celsius e.g. 32.5 °C
if unknown record 999

**Expected range** 20.00-42 00 °C

**Validation rule** Validation check if number exceeds 42.00 °C
Add warning if value not entered

## Maximum temperature recorded during first 24 hours

**Definition** The maximum temperature recorded during the first 24hours after start of sustained return of spontaneous circulation (sustained ROSC).

**Reason** Required to provide further detail about active core body temperature processes.



For peer review only - http://bmjopen.bmj.com/site/about/guidelines.xhtml

| | |
|---|---|
| **Format** | Record in degrees Celsius e.g. 37.5°C<br>if unknown record 999 |
| **Expected range** | 20.00-42.00°C |
| **Validation rule** | Validation check if number exceeds 42.00 °C<br>Add warning if value not entered<br>Add warning if maximum temperature <= minimum temperature |

## Comments

| | |
|---|---|
| **Definition** | Any additional information considered relevant to the dataset.<br>Text entered in this field may provide extra information about data entered elsewhere in a specific field in the dataset, or may provide extra information on the admission, which is not collected as part of the dataset.<br>No identifiers (patient, nurse, doctor, ICU, hospital) should be included in text data entered into this field.<br>As there is limited space in this field all text data should be kept to a minimum and be as concise as possible. Text data must not contain any punctuation except a period (full-stop) at the end of each data point. |
| **Reason** | No dataset specification covers all eventualities: to deal with this a text field has been included for comments/additional information. |
| **Format** | Free text |

## Form completed by

| | |
|---|---|
| **Definition** | Name of person completing form. |
| **Reason** | For local use only to assist with following up queries relating to completion of this form. |
| **Format** | Free text |



For peer review only - http://bmjopen.bmj.com/site/about/guidelines.xhtml

*NET-PACK 3: PICANet evaluation of post cardiac arrest care in kids*

*Please complete for* ***all PIC admissions following cardiac arrest*** *(include both out-of-hospital and in-hospital arrests)*

## Patient details *(or hospital label)*

**Family name**

**First name**

**Postcode**

**NHS/CHI/H&C number**

**Case note number**

**Date of birth** *(dd/mm/yyyy)*

__ __ / __ __ / __ __ __ __

## History at admission

***FOR OUT-OF-HOSPITAL CARDIAC ARREST ONLY:***

**Bystander CPR attempted?**

☐ Yes ☐ No ☐ Unknown

**Did CPR continue <u>after</u> arrival to the Emergency Department?**

☐ Yes ☐ No ☐ Unknown

***FOR IN <u>AND</u> OUT-OF-HOSPITAL CARDIAC ARREST:***

**First monitored cardiac rhythm during cardiac arrest**

☐ Asystole
☐ Sinus bradycardia < 60 bpm
☐ Pulseless electrical activity
☐ Ventricular fibrillation
☐ Ventricular tachycardia

*if rhythm detected by ECG*

☐ Shockable
☐ Non-shockable

*if rhythm detected by automated external defibrillator (AED)*

☐ No monitoring
☐ Unknown

**Time from observed cardiac arrest to start of sustained return of spontaneous circulation (ROSC)**

__ hours __ __ minutes

**Number of doses of epinephrine from initial resuscitation to start of period of sustained ROSC**

## Temperature management

**Core body temperature management during first 24 hours after sustained ROSC**

☐ Active Normothermia *(35 to 37.9 °C)*
☐ Active Therapeutic Hypothermia *(32 to <35 °C)*
☐ Other *(state below)*
☐ No active temperature control
☐ Unknown

**Duration of initial active temperature control management** *(if temperature actively managed)*

__ __ hours

**Minimum temperature recorded during first 24 hours**

__ __ . __ °C

**Maximum temperature recorded during first 24 hours**

__ __ . __ °C

## Comments

## Form completed by

## Contact us: picanet@leeds.ac.uk

**Sophie Butler**
*Project officer*
(0113) 343 8125
s.butler1@leeds.ac.uk

**Lee Norman**
*Database manager*
(0113) 343 8125
l.j.norman@leeds.ac.uk

**Caroline Lamming**
*Research nurse*
(0116) 252 5414
crl4@leicester.ac.uk

For peer review only - http://bmjopen.bmj.com/site/about/guidelines.xhtml

BMJ Open

# NEUROlogical Prognosis After Cardiac Arrest in Kids (NEUROPACK) study: protocol for a prospective multicentre clinical prediction model derivation and validation study in children after cardiac arrest.

| | |
|---|---|
| Journal: | *BMJ Open* |
| Manuscript ID | bmjopen-2020-037517.R1 |
| Article Type: | Protocol |
| Date Submitted by the Author: | 25-Jun-2020 |
| Complete List of Authors: | Scholefield, Barnaby; University of Birmingham College of Medical and Dental Sciences, Birmingham Acute Care Research Group; Birmingham Women and Children's NHS Foundation Trust, Paediatric Intensive Care Unit<br>Martin, James; University of Birmingham, Institute of Applied Health Research<br>Penny-Thomas, Kate; Birmingham Women and Children's NHS Foundation Trust, Paediatric Intensive Care Unit<br>Evans, Sarah; Birmingham Women and Children's NHS Foundation Trust, Paediatric Intensive Care Unit<br>Kool, Mirjam; University of Birmingham College of Medical and Dental Sciences, Birmingham Acute Care Research Group; Birmingham Women and Children's NHS Foundation Trust, Paediatric Intensive Care Unit<br>Parslow, Roger; University of Leeds, Leeds Institute for Data Analytics<br>Feltbower, Richard; University of Leeds, Leeds Institute for Data Analytics<br>Draper, Elizabeth; University of Leicester College of Medicine Biological Sciences and Psychology, Health Sciences<br>Hiley, Victoria; University of Leeds, Leeds Institute for Data Analytics<br>Sitch, Alice; University of Birmingham, Institute of Applied Health Research; University of Birmingham, NIHR Birmingham Biomedical Research Centre<br>Kanthimathinathan, Hari; Birmingham Women and Children's NHS Foundation Trust, Paediatric Intensive Care Unit ; University of Birmingham College of Medical and Dental Sciences, Birmingham Acute Care Research Group<br>Morris, Kevin; Birmingham Women’s and Children’s Hospital, Paediatric Intensive Care Unit; University of Birmingham, Institute of Applied Health Research<br>Smith, Fang; University of Birmingham College of Medical and Dental Sciences, Birmingham Acute Care Research Group |
| <b>Primary Subject Heading</b>: | Intensive care |
| Secondary Subject Heading: | Paediatrics |
| Keywords: | Paediatric intensive & critical care < INTENSIVE & CRITICAL CARE, |

| | |
|---|---|
| | Paediatric intensive & critical care < PAEDIATRICS, Protocols & guidelines < HEALTH SERVICES ADMINISTRATION & MANAGEMENT |
| | |

*I, the Submitting Author has the right to grant and does grant on behalf of all authors of the Work (as defined in the below author licence), an exclusive licence and/or a non-exclusive licence for contributions from authors who are: i) UK Crown employees; ii) where BMJ has agreed a CC-BY licence shall apply, and/or iii) in accordance with the terms applicable for US Federal Government officers or employees acting as part of their official duties; on a worldwide, perpetual, irrevocable, royalty-free basis to BMJ Publishing Group Ltd ("BMJ") its licensees and where the relevant Journal is co-owned by BMJ to the co-owners of the Journal, to publish the Work in this journal and any other BMJ products and to exploit all rights, as set out in our licence.*

*The Submitting Author accepts and understands that any supply made under these terms is made by BMJ to the Submitting Author unless you are acting as an employee on behalf of your employer or a postgraduate student of an affiliated institution which is paying any applicable article publishing charge ("APC") for Open Access articles. Where the Submitting Author wishes to make the Work available on an Open Access basis (and intends to pay the relevant APC), the terms of reuse of such Open Access shall be governed by a Creative Commons licence – details of these licences and which Creative Commons licence will apply to this Work are set out in our licence referred to above.*

*Other than as permitted in any relevant BMJ Author's Self Archiving Policies, I confirm this Work has not been accepted for publication elsewhere, is not being considered for publication elsewhere and does not duplicate material already published. I confirm all authors consent to publication of this Work and authorise the granting of this licence.*

For peer review only

**Protocol**

# NEUROlogical Prognosis After Cardiac Arrest in Kids (NEUROPACK) study: protocol for a prospective multicentre clinical prediction model derivation and validation study in children after cardiac arrest.


**Authors:**

Barnaby R Scholefield[1,2], James T Martin[3], Kate Penny-Thomas[2], Sarah Evans[2], Mirjam Kool[1,2], Roger Parslow[4], Richard Feltbower[4], Elizabeth S Draper[5], Victoria Hiley[4], Alice J Sitch[3,6], Hari Krishnan Kanthimathinathan[2,3], Kevin P Morris[2,3], Fang Gao Smith[1] On behalf of the NEUROPACK Investigators for the Paediatric Intensive Care Society-Study Group (PICS-SG)

[1] Birmingham Acute Care Research Group, Institute of Inflammation and Ageing, University of Birmingham, UK
[2] Paediatric Intensive Care Unit, Birmingham Women and Children's Hospital NHS Trust
[3] Institute of Applied Health Research, University of Birmingham, UK
[4] Leeds Institute for Data Analytics, School of Medicine, University of Leeds
[5] Department of Health Sciences, University of Leicester
6 NIHR Birmingham Biomedical Research Centre, University Hospitals Birmingham NHS Foundation Trust and University of Birmingham, UK

Corresponding author:
Dr Barnaby R Scholefield, Birmingham Acute Care Research Group, Institute of Inflammation and Ageing, University of Birmingham, UK
b.scholefield@bham.ac.uk


For peer review only

# Abstract:

**Introduction:**

Currently, we are unable to accurately predict mortality or neurological morbidity following resuscitation after paediatric out of hospital (OHCA) or in-hospital (IHCA) cardiac arrest. A clinical prediction model may improve communication with parents and families and risk stratification of patients for appropriate post-cardiac arrest care. This study aims to the derive and validate a clinical prediction model to predict, within one hour of admission to the paediatric intensive care unit (PICU), neuro-developmental outcome at three months after paediatric cardiac arrest.

**Methods and analysis:**

A prospective study of children (age: >24 hours and <16 years), admitted to one of the 27 participating PICUs in the UK and Ireland, following an OHCA or IHCA. Patients are included if requiring more than one minute of cardiopulmonary resuscitation and mechanical ventilation at PICU admission Children who had cardiac arrests in PICU or neonatal intensive care unit will be excluded. Candidate variables will be identified from data submitted to the Paediatric Intensive Care Audit Network (PICANet) registry. Primary outcome is neuro-developmental status, assessed at three months by telephone interview using the Vineland Adaptive Behavioural Score II questionnaire. A clinical prediction model will be derived using logistic regression with model performance and accuracy assessment. External validation will be performed using the Therapeutic Hypothermia After Pediatric Cardiac Arrest (THAPCA) trial dataset. We aim to identify 370 patients, with successful consent and follow up of 150 patients. Patient inclusion started 1st January 2018 and inclusion will continue over 18 months.

**Ethics and dissemination:**

Ethical review of this protocol was completed by 27th September 2017 at the Wales Research Ethics Committee 5, 17/WA/0306. The results of this study will be published in peer reviewed journals and presented in conferences.


**Trial Registration Number:** ClinicalTrials.gov NCT03574025

**Keywords:** Observational; Paediatric; Cardiac Arrest; PICU; Prognostication

**Word Count Abstract:** 294

**Word Count Main text**: 2835



For peer review only - http://bmjopen.bmj.com/site/about/guidelines.xhtml

# Article Summary

## Strengths and limitations of this study

- This protocol has followed the international recommended TRIPOD guidelines for the derivation and validation of a clinical prediction model of neurodevelopmental outcome after paediatric cardiac arrest.
- A nationwide study which will efficiently combine routinely collected data through the existing, high quality, Paediatric Intensive Care Audit Network (PICANet) database and a bespoke research database.
- Personalised recruitment and local follow up will aim to maximise participant retention.
- The low incidence and wide variety of causes of paediatric cardiac arrest may restrict number of available patients and are potential limitations in prospective prognostic research in this population.
- Baseline neurodevelopmental status of patients will only be allocated retrospectively using the Pediatric Cerebral Performance Category (PCPC) tool.

# Introduction

**Paediatric cardiac arrest**

Paediatric cardiac arrest (CA) is an uncommon but potentially catastrophic event for both children and their families. Cardiac arrest is defined as the cessation of cardiac mechanical activity occurring with absence of signs of circulation. Approximately 1500 infants or children per year suffer a cardiac arrest in the United Kingdom (UK) and Republic of Ireland (RoI) with between 250-350 admitted to a paediatric intensive care unit (PICU) for post resuscitation care.[1] Survival to PICU discharge for this population is achieved in 35-45% patients admitted to PICU after an OHCA and 45-55% after IHCA. However, 50% of survivors are estimated to have ongoing neuro-developmental disabilities despite advances in post-cardiac arrest management.[2 3] The high mortality and morbidity rates are often associated with the degree of brain injury from the hypoxic-ischaemic insult at the time of cardiac arrest.

**Prognostication after cardiac arrest**

Clinicians are currently unable to accurately predict survival with a good neuro-developmental outcome after cardiac arrest with any certainty due to a lack of data.[4-6] Clinicians can be pessimistic, optimistic or unnecessarily ambiguous in their predictions, and this affects the clarity of communication with families and the implementation of on-going treatment plans.[4] Improved prognostication is therefore a high priority for parents of children who have suffered a CA. In addition, early stratification of patients who may benefit from critical care interventions would also be a significant advancement in their treatment[7 8] and has been lacking in major studies to date.[2 3]



For peer review only - http://bmjopen.bmj.com/site/about/guidelines.xhtml

Several prognostic factors are associated with survival following paediatric CA, such as patient age and pre-existing co-morbidities[9], cardiac arrest characteristics (location, initial cardiac arrest rhythm, duration of cardiac arrest, presence and actions of bystanders,[9 10] physiological observations (e.g. pupillary response, blood lactate, systolic blood pressure)[10-12] and specific medical interventions.[12 13] However, studies examining prognostic factors for good neuro-developmental outcome are much less frequent.

The importance and weighting of these factors in prognosis decision making is complex and in 2010 the International Liaison Committee On Resuscitation (ILCOR) consensus statement identified a significant gap in knowledge in prognostic modelling with children[5] with no additional 'high quality' data to inform the 2015 guidance.[14]

**Rationale for study**
Accurate early prediction of neuro-developmental outcomes may reduce uncertainty and improve communication with families. It may also provide better risk-stratification for clinical trials and individualised treatment of patients. Furthermore, we aim to gain a better understanding of the epidemiology and neurodevelopmental outcomes of children after CA in the UK and RoI.

# Methods and analysis:

**Study Aims:**
The aim of the NEUROPACK study is to 1) derive a clinical prediction model using key factors prospectively collected from a cohort of patients, available within the first hour of PICU admission after paediatric cardiac arrest to predict good neuro-developmental outcome at three months, 2) externally validate the clinical prediction model using an existing paediatric cardiac arrest dataset and 3) describe the current epidemiology of cardiac arrest cases in the United Kingdom (UK) and Republic of Ireland (RoI).

**Study Design:**
This study is a multi-centre, nationwide, prospective observational study combining both registry and cohort data. See Figure 1 for study overview.

**Setting:**
Patients will be enrolled from 24 PICUs within the UK and RoI. All study sites admit infants and children following cardiac arrest and routinely submit audit data to the Paediatric Intensive Care Audit Network (PICANet) registry.

**Ongoing PICU Registry: PICANet and NET-PACK 3:**
Since 2002, PICANet has prospectively collected demographic, diagnostic, and interventional data along with PICU survival outcomes for patients admitted to PICUs in England and Wales and now collects data for patients across the United Kingdom and RoI.[15] This includes severity of illness variables to build the Paediatric Index of Mortality risk-adjustment models.[16]

PICANet is also conducting an ongoing customised data collection of post-cardiac arrest management: PICANet Post Arrest Care in Kids (NET-PACK 3) with data definition and data collection form (Supplemental material 1 & 2). NET-PACK 3 customised data collection includes resuscitation variables available within a few hours of the CA. Data are either collected within one hour of



For peer review only - http://bmjopen.bmj.com/site/about/guidelines.xhtml
For peer review only

admission onto PICU or within one hour of the attendance at the patient's bedside of a specialist paediatric critical care team (e.g. a specialised retrieval team travels to another hospital without a PICU). These variables include: 1) attempted bystander cardiopulmonary resuscitation (CPR), 2) duration of CPR, 3) requirement of CPR after arrival at emergency department, 4) number of doses of epinephrine (adrenaline) required and 5) initial presenting cardiac rhythm. These factors were chosen to comply with Utstein style CA reporting guidelines[17 18]. PICANet collects survival to PICU discharge outcome data for all admissions.

## Eligibility for NEUROPACK:

### Inclusion

All patients aged 24 hours up to 16th birthday admitted to PICU after OHCA or IHCA will be included. Cardiac arrest will be defined as requiring > 1minute CPR. Patients will be included if they require invasive (e.g. via endotracheal or tracheostomy) mechanical ventilation at PICU admission.

### Exclusion

Exclusion criteria include cardiac arrests occurring within a PICU or neonatal intensive care unit. For children who survive to PICU discharge we will exclude patients where the local clinical team at participating sites feel inclusion is inappropriate and/or parent/guardian or family member of children are unable to understand the telephone questionnaires for neuro-developmental outcome assessments in English. All patients under the age of 24 hours will be excluded due to potentially different aetiology of CA related to birth events.

#### Identification and screening

Patients for the NEUROPACK study will be identified via entry into the PICANet database and by local researchers at each site screening PICU admissions daily. 'Cardiac arrest preceding ICU admission – out of hospital or in-hospital' is a specific high risk category in the Paediatric Index of Mortality (PIM-3) risk-adjustment model and is recorded within one hour of PICU admission, or within one hour of the attendance at the patient's bedside of a specialist paediatric critical care team.[16]

### Recruitment for neuro-developmental outcome assessment

Parent/guardians of CA patients who are expected to survive to three months following CA will be approached by local research staff, trained in Good Clinical Practice, to consent for telephone questionnaire at three months post CA.

This is a very sensitive and difficult time for parents and guardians. The approach to parents or guardians of critical ill children for recruitment to the NEUROPACK study will therefore be handled sensitively. Local researchers will be trained to identify the appropriate time to consent, utilise passive information giving to reduce burden of information (e.g. Ethics committee-approved posters displayed in family rooms) and liaise with the medical team managing the patient to acknowledge ongoing clinical management issues. Local site investigator (or delegate) will re-contact parents or guardians at two months following CA to ascertain continued involvement in the study and to confirm ongoing contact details.

### Potential predictive factors collected

Potential candidate variables for the NEUROPACK clinical prediction model have been selected from the existing clinical prediction models for survival.[6 11 14] Final candidate variable selection will follow assessment of statistical modelling interaction and practicality of collecting variables in a timely fashion at the bedside by clinicians.



For peer review only - http://bmjopen.bmj.com/site/about/guidelines.xhtml

**Data collections**

The ongoing NET-PACK 3 customised data collection and PICANet data collection for the PIM3 risk of mortality will be the data source for all the candidate variables in the NEUROPACK study. Linkage of individual patient NET-PACK 3 data with the collected neuro-developmental outcome will be carried out for consented patients only. Pseudonymised data from NET-PACK 3 customised data collection and PICANET will be used for patients who die or for patients who survive and consent for follow up assessment is not available.

## Primary and secondary outcomes

### Primary outcome

The primary outcome is survival with a good neuro-developmental outcome at three months post event. Good neuro-developmental outcome is defined as a Vineland Adaptive Behaviour Scales 2nd edition (VABS-II) score of ≥ 70.[19]

### Primary outcome assessment

The VABS-II was designed as a caregiver report measure to assess communication, daily living, social, and motor domains of adaptive behaviour.[19] This tool can be used across the entire paediatric age range (0 to 16years) and requires a short interview which can be via telephone. VABS-II is sensitive to neurological injury and has been used successfully in paediatric neuro-critical care studies.[2] VABS-II has a normal mean value score of 100 (standard deviation of 15). Good neuro-developmental outcome is defined as a score of ≥ 70. Poor outcome is a composite score of VABS-II <70 and death. The chief investigator or the lead research nurse at the Central Research Centre (Birmingham Women & Children's NHS Foundation Trust, UK) will conduct all assessments. At the time of outcome assessment, the assessor will remain blinded to the clinical prediction model and component variables.

### Secondary outcomes

Paediatric cerebral performance category (PCPC) and paediatric overall performance category (POPC) at three months and change in PCPC and POPC score from baseline.[20] Survival to PICU discharge and three months post cardiac arrest.

### Secondary outcome assessment

PCPC and POPC scale can be calculated by a short questionnaire conducted at the 3 month follow up interview for consented patients. A baseline (pre-cardiac arrest) PCPC and POPC will also retrospectively ascertained at the three month follow up. PCPC and POPC have been recommended for reporting in all paediatric CA studies. They score 1 to 6 (1: normal, 2: mild disability, 3: moderate disability, 4: severe disability, 5: vegetative state or coma and 6: death). They provide less detail but correlate reasonably well with VABS II.[21] This will allow comparison with other CA studies. Good neurodevelopmental outcome will be defined as PCPC score of 1 to 3 or no change from baseline. Poor outcome will be defined as a score of 4 or more, including death. Three months follow up time point is chosen following the International Liaison Committee On Resuscitation (ILCOR), core-outcome set for adults after cardiac arrest (COSCA) recommendation[22] and demonstration of minimal change between three and 12 month following cardiac arrest.[23]

## Statistical consideration

### Data analysis plan



For peer review only - http://bmjopen.bmj.com/site/about/guidelines.xhtml

The data will be manually reviewed for errors, missing data and outliers before analysis. Extreme values will be set to missing if they are deemed unlikely, based on their validity range. Descriptive analysis of the data will be reported. Continuous variables will be reported as either median and interquartile range (IQR) or mean and standard deviation (SD) based on the distribution. Categorical variables will be described in numbers, percentages or both.

**Table 1. Patient and cardiac arrest characteristics**

| **Table 1. Patient and cardiac arrest characteristics** |
|---|
| **Patient Demographic** |
| • **Age in years^** |
| • **Presence of PIM-3 'high risk' co-morbidities*[16]** |
| **Cardiac arrest characteristics and interventions:** |
| • **Location of cardiac arrest (IH & OHCA)*** <br> ***OHCA is assigned if chest compressions were initiated before hospital arrival*** |
| • **Aetiology of arrest (cardiac & non-cardiac)*** |
| • **Duration of cardiopulmonary resuscitation^** <br> • **Continuation of cardiopulmonary resuscitation after Emergency Department arrival (for OHCA only)*.** |
| • **Bystander cardiopulmonary resuscitation*** |
| • **Initial cardiac rhythm recorded during CA (shockable & non-shockable)*** |
| • **Doses of epinephrine (adrenaline) during cardiopulmonary resuscitation^** |
| • **Use of continuous vasoactive infusions within one hour of PICU admission*** |
| **Service characteristics:** |
| • **Requirement of inter-hospital transfer prior to PICU admission*** |
| • **Time of arrest day (07:00 – 18:59) or night (19:00 – 06:59)*** |
| **Physiological variables:** <br> ***Measured for PIM-3 calculation: within one hour of PICU admission or within one hour of the attendance at the patient's bedside of a specialist paediatric critical care team*** |
| • **Systolic blood pressure^** |
| • **Pupillary reaction to light (greater than 3mm and both fixed & other) *** |
| • **Blood lactate level^** |
| ***categorical data, ^continuous data.** <br> ***PIM-3: Paediatric Index of Mortality 3 score, IH: in-hospital, OHCA: out-of-hospital cardiac arrest*** |

**Sample Size**

To reduce problematic bias and improve precision we aim for at least 10 events per variable considered for multivariable modelling[24]. Following pilot data collection, we calculate 250 CA patients per year are admitted to 27 UK and RoI PICUs, 125 (50%) will survive to PICU discharge and 70 (30%) per year will survive with good neuro-developmental outcome. To test 7 variables we estimate a requirement of 70 events (e.g. patients with good neuro-developmental outcome). 100% of non-survivors will be included (included in PICANet and NET-PACK 3 audit database). We anticipate 80% recruitment and consent rate of remaining survivors. We therefore require data collection over an 18-month period to recruit 370 patients. We anticipate that this would ensure successful consent and follow up of 150 patients, of whom 75 patients are estimated to have a good neuro-developmental outcome.**Statistical methods for developing a prognostic model**

We will develop a prognostic model using logistic regression analysis of candidate variables and a good neuro-developmental outcome as the primary outcome variable. Multiple imputation (using



For peer review only - http://bmjopen.bmj.com/site/about/guidelines.xhtml

chained equations) will be used for any variables with missing data considered in the model. Auxiliary variables will be used to aid the imputation. The number of imputed data sets used will be equal to the fraction of missing data[25].

Table 1 lists all candidate variables. Those variables deemed to be clinically important will be forced into the final model. Candidate variables will be retained if they benefit the model. The process will begin by fitting the full model and then performing backwards elimination, with a conservative significance level of 0.157.[26] For categorical variables, the category with the lowest *p*-value will dictate whether the variable is included in the final model.

All continuous variables will be left in their raw form to ensure no data was lost through dichotomisation or categorisation. It will be initially assumed that variables follow a linear trend, before fractional polynomials will be considered using the following powers: -2, -1, -0.5, natural logarithm, 0.5, 1, 2, and 3. A *p*-value <0.001 will be required to use a fractional polynomial rather than assuming a linear trend.[27] The use of fractional polynomials will also be considered for all continuous variables eliminated from the model to check whether this changes their inclusion status.

**Assessment of prognostic model performance**

Assessment of the fitted model will be achieved by estimating calibration and discrimination. A calibration plot will be produced by plotting the observed risk against the predicted risk and the calibration slope calculated. We expect the slope should be approximately 1 as the model developed will be developed using this data. To judge discrimination, the area under the receiver operating curve (equivalent to the c-statistic) and the R squared statistic will be calculated.

**Internal validation of the prognostic model**

The model will be internally validated using bootstrap methods. The original data will be used to generate 100 bootstrapped data sets. Each one of these bootstrapped data sets will then be used to develop a prognostic model in the same way as the original model. Estimates of performance (c-statistic and calibration slope) will be obtained from the model fitted using each of the bootstrapped data sets. The estimates obtained from the bootstrapped data sets will be averaged and subtracted from the estimates from the original model to estimate optimism and provide optimism-adjusted performance statistics.

**Final prognostic model**

The optimised adjusted calibration slope will then be used as a uniform shrinkage factor. Each of the coefficients from the original model will be adjusted for by multiplying by the shrinkage factor. The intercept will also be adjusted to ensure calibration-in-the-large, the average predicted probability, is the same as the average observed probability.

**Secondary analysis**

Using the secondary outcomes, we will repeat the steps above to create a supplemental final prognostic model, for survival to PICU and survival to three months. In addition we will create a prognostic model for good neurodevelopmental outcome using POPC and PCPC outcome scores.

There is a potential for survivors to decline consent, be lost to follow up, or fulfil the exclusion criteria into the NEUROPACK study and therefore there is a risk that the survival subgroup may be



For peer review only - http://bmjopen.bmj.com/site/about/guidelines.xhtml

biased. We plan to undertake sensitivity analyses by 1) imputing missing VABS II score for survivors using their known PICANet and NETPACK 3 data, 2) assume all survivors without a neurodevelopmental score had a VABS II score ≥ 70 and 3) assume all survivors without a neurodevelopmental score had a VABS II score <70, to ascertain impact of this group on the final prognostic model.

In addition, due to the limitations of not having a baseline VABS II score, we will also perform a secondary analysis using VABS II score ≥ 70 as the good neurodevelopmental outcome for a subgroup of patients with a known baseline PCPC score 1-3. This will allow comparison of the final prognostic model for all patients and the subgroup with known good neurodevelopment outcome at baseline.

**External Validation of the NEUROPACK prognostic score**

As part of the process of ensuring a prediction model is considered clinically useful, it must be validated in an external dataset.[28] We aim to do this by validating the NEUROPACK prognostic model in the publically accessible dataset for the Therapeutic Hypothermia After Pediatric Cardiac Arrest (THAPCA) OHCA and IHCA randomised controlled trials in the National Institute for Health Biolincc repository (Http://biolincc.nhlbi.nih.gov).[2 3] The sample size of the dataset to be used for external validation should be sufficient to provide reliable and accurate results. To externally validate the model, predictions of risk for each patient in the external validation dataset are made, and performance statistics, such as the C-statistic, are calculated in the same manner as described earlier.

# Patient and Public Involvement:

Given the sensitive and emotive nature of the NEUROPACK study, and the need for active parent and family engagement throughout, a patient advisory group, consisting of parents with experience of critical illness and death in children, and the Clinical Research Network: Children young person's advisory group (a sub group of the Generation R group aged 9-17yrs) have been consulted in designing the protocol, the informational material to support the intervention, and to understand the burden of the intervention from the patient's perspective. At the end of the study, the patient advisory group will be consulted on findings and contribute to the dissemination plan.

# Ethics and dissemination:

PICANet has ethical approval as a research database granted by the East Midlands, Derby Research Ethics Committee (ref 18/EM/0267) and NHS Health Research Authority Confidentiality Advisory Group approval (ref PIAG 4-07/(c)2002) to collect personally identifiable data without consent. The PICANet Clinical Advisory Group has approved pseudonymised sharing of PICANet audit data for the NEUROPACK study and Data Sharing Agreements will be established with the data controllers for the PICANet dataset prior to the release of de-identified PICANet and NET-PACK 3 data. Quality control of NET-PACK 3 customised data collection, data definitions and data collection is performed by the PICANet team.

Regional Ethics Committee (REC) permission has been obtained (Wales Research Ethics Committee 5, 17/WA/0306). This permits the ethical approach and consent of parents/guardians of eligible



For peer review only - http://bmjopen.bmj.com/site/about/guidelines.xhtml

children who are likely to survive to 3 months following CA to enable telephone VABS-II assessment and identified data-linkage and sharing with PICANet and NET-PACK3 data.

We aim to publish the results in peer reviewed journals and present at relevant national and international conferences.


# Acknowledgements:

We would like to thank all participating sites for their contribution to data collection. We thank the NEUROPACK Principle Investigators from the Paediatric Intensive Care Society Study Group (PICS-SG) listed below:

1. Alder Hey Children's Hospital, Liverpool, UK. Dr Kent Thorburn
2. Birmingham Women and Children's Hospital NHS Foundation Trust, UK. Dr Barnaby Scholefield
3. Bristol Royal Hospital for Children, UK. Dr Rohit Saxena
4. Freeman Hospital, Newcastle, UK. Dr Yamuna Thiru
5. Glasgow Royal Hospital for Children, UK. Dr Richard Levin
6. Great North Children's Hospital, Newcastle, UK. Dr Rachel Agbecko
7. Great Ormond Street Hospital, London, UK. Dr Timothy Thiruchelvam & Dr Sophie Skellett
8. Imperial College Healthcare NHS Trust, London, UK. Dr David Inwald.
9. John Radcliff Hospital, Oxford, UK. Dr James Weitz
10. Kings College Hospital NHS Foundation Trust, London, UK. Dr Akash Deep
11. Leeds Children's Hospital, UK. Dr Sian Cooper
12. Leicester Children's Hospital, UK. Dr Peter Barry
13. Nottingham Children's Hospital, UK. Dr Patrick Davies
14. Our Ladies Children's Hospital, Crumlin, Dublin, Republic of Ireland. Dr Cormac Breatnach.
15. Royal Brompton Hospital, London, UK. Dr Sandra Gala-Peralta
16. Royal Hospital for Sick Children, Edinburgh, UK. Dr Milly Lo.
17. Royal Manchester Children's Hospital, UK. Dr Rachael Barber
18. Royal Victoria Hospital, Belfast, UK. Dr Stewart Reid.
19. Sheffield Children's NHS Foundation Trust, UK. Dr Rum Thomas.
20. Southampton General Hospital, UK. Dr John Pappachan.
21. St George's Hospital, London, UK. Dr Buvana Dwarakanathan.
22. The Noah's Ark Children's Hospital for Wales, UK. Cardiff. Dr Siva Oruganti.
23. The Royal London Hospital, UK. Dr Kalai Sadasivam.
24. University Hospitals of North Midlands, Stoke on Trent, UK. Dr Mark Bebbington


For peer review only



For peer review only - http://bmjopen.bmj.com/site/about/guidelines.xhtml

# References

1. Scholefield BR, Gao F, Duncan HP, et al. Observational study of children admitted to United Kingdom and Republic of Ireland Paediatric Intensive Care Units after out-of-hospital cardiac arrest. *Resuscitation* 2015 doi: 10.1016/j.resuscitation.2015.07.011 [published Online First: 2015/07/25]
2. Moler FW, Silverstein FS, Holubkov R, et al. Therapeutic Hypothermia after Out-of-Hospital Cardiac Arrest in Children. *The New England journal of medicine* 2015 doi: 10.1056/NEJMoa1411480 [published Online First: 2015/04/29]
3. Moler FW, Silverstein FS, Holubkov R, et al. Therapeutic Hypothermia after In-Hospital Cardiac Arrest in Children. *The New England journal of medicine* 2017;376(4):318-29. doi: 10.1056/NEJMoa1610493 [published Online First: 2017/01/25]
4. Fallowfield L, Jenkins V. Communicating sad, bad, and difficult news in medicine. *Lancet (London, England)* 2004;363(9405):312-9. doi: 10.1016/s0140-6736(03)15392-5 [published Online First: 2004/01/31]
5. Kleinman ME, de Caen AR, Chameides L, et al. Part 10: Pediatric basic and advanced life support: 2010 International Consensus on Cardiopulmonary Resuscitation and Emergency Cardiovascular Care Science With Treatment Recommendations. *Circulation* 2010;122(16 Suppl 2):S466-515. doi: 10.1161/CIRCULATIONAHA.110.971093 [published Online First: 2010/10/22]
6. Phillips RS, Scott B, Carter SJ, et al. Systematic Review and Meta-Analysis of Outcomes after Cardiopulmonary Arrest in Childhood. *PloS one* 2015;10(6):e0130327. doi: 10.1371/journal.pone.0130327 [published Online First: 2015/06/25]
7. Kent DM, Hayward RA. Limitations of applying summary results of clinical trials to individual patients: the need for risk stratification. *Jama* 2007;298(10):1209-12. doi: 10.1001/jama.298.10.1209 [published Online First: 2007/09/13]
8. Hingorani AD, Windt DA, Riley RD, et al. Prognosis research strategy (PROGRESS) 4: stratified medicine research. *BMJ (Clinical research ed)* 2013;346:e5793. doi: 10.1136/bmj.e5793 [published Online First: 2013/02/07]
9. Atkins DL, Everson-Stewart S, Sears GK, et al. Epidemiology and outcomes from out-of-hospital cardiac arrest in children: the Resuscitation Outcomes Consortium Epistry-Cardiac Arrest. *Circulation* 2009;119(11):1484-91.
10. Moler FW, Donaldson AE, Meert K, et al. Multicenter cohort study of out-of-hospital pediatric cardiac arrest. *Crit Care Med* 2011;39(1):141-9. doi: 10.1097/CCM.0b013e3181fa3c17 [published Online First: 2010/10/12]
11. Scholefield BR, Gao F, Duncan HP, et al. Observational study of children admitted to United Kingdom and Republic of Ireland Paediatric Intensive Care Units after out-of-hospital cardiac arrest. *Resuscitation* 2015;97:122-8. doi: 10.1016/j.resuscitation.2015.07.011 [published Online First: 2015/07/25]
12. Meert KL, Telford R, Holubkov R, et al. Pediatric Out-of-Hospital Cardiac Arrest Characteristics and Their Association With Survival and Neurobehavioral Outcome. *Pediatric critical care medicine : a journal of the Society of Critical Care Medicine and the World Federation of*

For peer review only



For peer review only - http://bmjopen.bmj.com/site/about/guidelines.xhtml

*Pediatric Intensive and Critical Care Societies* 2016;17(12):e543-e50. doi: 10.1097/pcc.0000000000000969 [published Online First: 2016/09/30]
13. Rodriguez-Nunez A, Lopez-Herce J, Garcia C, et al. Effectiveness and long-term outcome of cardiopulmonary resuscitation in paediatric intensive care units in Spain. *Resuscitation* 2006;71(3):301-9.
14. American Heart Association. Post ROSC predictive factors for survival in infants and children. 2015. https://volunteer.heart.org/apps/pico/Pages/PublicComment.aspx?q=813 (accessed 1st June 2015).
15. PICANET. The Paediatric Intensive Care Audit Network (PICANet) Project protocol. 2008. https://www.picanet.org.uk/wp-content/uploads/sites/25/2020/01/PICANet-project-protocol-v4.0_2018_08Aug_08.pdf (accessed 01 July 2019).
16. Straney L, Clements A, Parslow RC, et al. Paediatric index of mortality 3: an updated model for predicting mortality in pediatric intensive care*. *Pediatric critical care medicine : a journal of the Society of Critical Care Medicine and the World Federation of Pediatric Intensive and Critical Care Societies* 2013;14(7):673-81.
17. Perkins GD, Jacobs IG, Nadkarni VM, et al. Cardiac Arrest and Cardiopulmonary Resuscitation Outcome Reports: Update of the Utstein Resuscitation Registry Templates for Out-of-Hospital Cardiac Arrest: A Statement for Healthcare Professionals From a Task Force of the International Liaison Committee on Resuscitation (American Heart Association, European Resuscitation Council, Australian and New Zealand Council on Resuscitation, Heart and Stroke Foundation of Canada, InterAmerican Heart Foundation, Resuscitation Council of Southern Africa, Resuscitation Council of Asia); and the American Heart Association Emergency Cardiovascular Care Committee and the Council on Cardiopulmonary, Critical Care, Perioperative and Resuscitation. *Resuscitation* 2015;96:328-40. doi: 10.1016/j.resuscitation.2014.11.002 [published Online First: 2014/12/02]
18. Zaritsky A, Nadkarni V, Hazinski MF, et al. Recommended guidelines for uniform reporting of pediatric advanced life support: the pediatric Utstein style. A statement for healthcare professionals from a task force of the American Academy of Pediatrics, the American Heart Association, and the European Resuscitation Council. *Pediatrics* 1995;96(4 Pt 1):765-79. [published Online First: 1995/10/01]
19. Sparrow S, Cicchetti D, Balla D. Vineland Adaptive Behavior Scales (2nd edition). Circle Pines, MN: AGS Publishing 2005.
20. Fiser DH, Long N, Roberson PK, et al. Relationship of pediatric overall performance category and pediatric cerebral performance category scores at pediatric intensive care unit discharge with outcome measures collected at hospital discharge and 1- and 6-month follow-up assessments. *Critical care medicine* 2000;28(7):2616-20.
21. Slomine BS, Silverstein FS, Christensen JR, et al. Neuropsychological Outcomes of Children 1 Year After Pediatric Cardiac Arrest: Secondary Analysis of 2 Randomized Clinical Trials. *JAMA Neurol* 2018;75(12):1502-10. doi: 10.1001/jamaneurol.2018.2628 [published Online First: 2018/09/23]
22. Haywood K, Whitehead L, Nadkarni VM, et al. COSCA (Core Outcome Set for Cardiac Arrest) in Adults: An Advisory Statement From the International Liaison Committee on Resuscitation. *Resuscitation* 2018;127:147-63. doi: 10.1016/j.resuscitation.2018.03.022 [published Online First: 2018/05/01]
23. Slomine BS, Silverstein FS, Christensen JR, et al. Neurobehavioural outcomes in children after In-Hospital cardiac arrest. *Resuscitation* 2018;124:80-89. doi: 10.1016/j.resuscitation.2018.01.002 [published Online First: 2018/01/07]
24. Peduzzi P, Concato J, Kemper E, et al. A simulation study of the number of events per variable in logistic regression analysis. *J Clin Epidemiol* 1996;49(12):1373-9. [published Online First: 1996/12/01]



For peer review only - http://bmjopen.bmj.com/site/about/guidelines.xhtml

25. White IR, Royston P, Wood AM. Multiple imputation using chained equations: Issues and guidance for practice. *Stat Med* 2011;30(4):377-99. doi: 10.1002/sim.4067 [published Online First: 2011/01/13]
26. Sauerbrei W. The Use of Resampling Methods to Simplify Regression Models in Medical Statistics. *Journal of the Royal Statistical Society Series C (Applied Statistics)* 1999;48(3):313-29.
27. Royston P, Altman DG. Regression Using Fractional Polynomials of Continuous Covariates: Parsimonious Parametric Modelling. *Journal of the Royal Statistical Society Series C (Applied Statistics)* 1994;43(3):429-67. doi: 10.2307/2986270
28. Collins GS, de Groot JA, Dutton S, et al. External validation of multivariable prediction models: a systematic review of methodological conduct and reporting. *BMC Med Res Methodol* 2014;14:40. doi: 10.1186/1471-2288-14-40 [published Online First: 2014/03/22]

For peer review only

# Authors' contributions

BS: initiated the collaborative project, designed the study, designed data collection tools, wrote the statistical analysis plan, drafted and revised the paper. He is guarantor. JM: wrote the statistical analysis plan, revised the paper. KP-T designed data collection tools, revised the paper. AS: wrote the statistical analysis plan, revised the paper. RP, RF, ESD and VH: advised on PICANet data utility, monitor's data collection for PICANET and revised the paper. SE, MK, HKK, KM, FGS: designed the study, drafted and revised the paper.

# Funding statement

Dr Barnaby Scholefield is funded by a National Institute for Health Research (NIHR) Clinician Scientist Fellowship (NIHR-CS-2015-15-016) for this research project.

# Consent

All patients and families will provide written informed consent/assent and will have the ability to withdraw at any time without explanation.

# Disclaimer

This publication presents independent research funded by the National Institute for Health Research (NIHR). The views expressed are those of the author(s) and not necessarily those of the NHS, the NIHR or the Department of Health and Social Care

# Conflicts of interests statement

None declared.



For peer review only - http://bmjopen.bmj.com/site/about/guidelines.xhtml

# Supplemental Material.

## 1. NETPACK 3 data definitions

https://www.picanet.org.uk/wp-content/uploads/sites/25/2018/05/NET-PACK-3-Data-Definitions-v1.1.pdf

## 2. NETPACK 3 data collection form

https://www.picanet.org.uk/wp-content/uploads/sites/25/2018/05/PICANet-form-custom-audit-NET-PACK-3-v1.4-June-2017.pdf

## Figure 1 Caption:

NEUROPACK Study Overview: Population, data collection tools and primary outcome



For peer review only - http://bmjopen.bmj.com/site/about/guidelines.xhtml

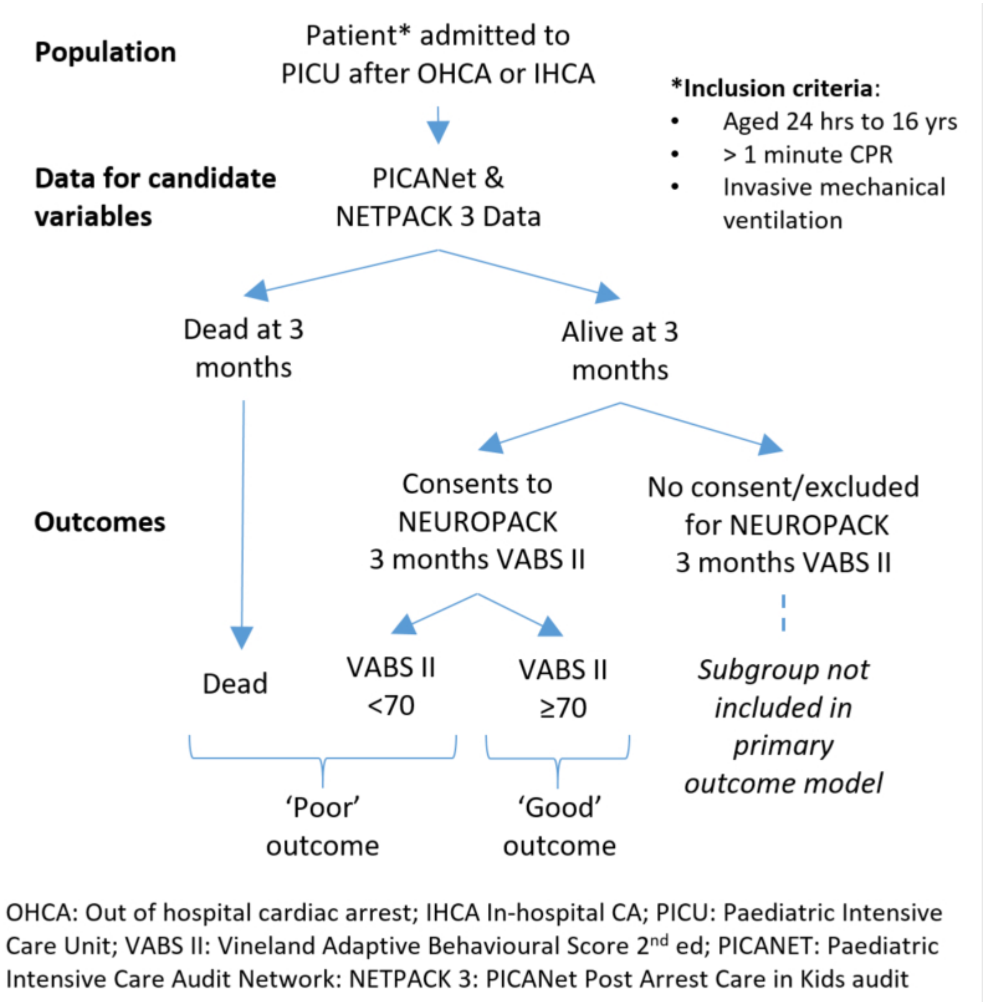


NEUROPACK Study Overview: Population, data collection tools and primary outcome

# PICANet Custom Audit Definitions

# NET-PACK 3

Version 1.1 (May 2017)

For peer review only

# Contents

Introduction ........ 2
Background ........ 2
References ........ 2
Data collection method ........ 3
Patient details ........ 6
Family name or Surname ........ 6
First name ........ 6
Postcode ........ 6
NHS, CHI or H&C number ........ 7
Case note number ........ 7
Date of birth ........ 7
History at admission ........ 8
Bystander Cardiopulmonary Resuscitation (CPR) Attempted? ........ 8
Cardiopulmonary Resuscitation continued after arrival to the Emergency Department? ........ 8
First monitored cardiac rhythm during cardiac arrest ........ 9
Time from observed cardiac arrest to start of sustained return of spontaneous circulation (ROSC) 9
Number of doses of epinephrine from initial resuscitation to start of period of sustained ROSC ... 10
Temperature management ........ 10
Core body temperature management planned during first 24 hours after sustained ROSC ........ 10
Duration of initial active temperature control management ........ 11
Minimum temperature recorded during first 24 hours ........ 11
Maximum temperature recorded during first 24 hours ........ 11
Comments ........ 12
Form completed by ........ 12

For peer review only

# Introduction

## Background

The NET-PACK 3 Custom Audit - PICANet evaluation of Post cardiac Arrest Care in Kids, is a re-audit of patient management after cardiac arrest in UK and Irish PICUs.

Between June 2014 and December 2015, in collaboration with Dr Barney Scholefield (Chief Investigator) at Birmingham Children's Hospital PICU and the Paediatric Intensive Care Society (PICS), PICANet performed the NET-PACK **2** custom audit in 29 UK and Irish PICUs.

Additional data was collected about post cardiac arrest management for either out-of-hospital or in-hospital cardiac arrest prior to PICU admission in 400 infants and children. Eight resuscitation variables available at the time of PICU admission and the early proposed post cardiac arrest temperature management plans were collected. The key findings will be published in detail shortly.

Importantly wide variation in PICU post-arrest management has been identified and also opportunities to stratify the cardiac arrest population for targeted treatments.

NET-PACK 3 has been designed to investigate the impact and compliance with the new International guidance and research data on post-arrest care as part of the PICANet clinical audit function. In December 2015 the International Liaison Committee on Resuscitation (ILCOR) published up-to-date guidance on Paediatric Advanced Life Support and post-cardiac arrest management (1). In addition two large randomised controlled trials of targeted temperature management after paediatric cardiac arrest have been published (2, 3). The primary objective of the NET-PACK 3 custom audit will be to assess whether targeted temperature management (TTM) is used, the dose of TTM (duration and temperature) following the ILCOR 2015 guidance and trial recent publications and the effect on survival outcome. In addition the NET-PACK 3 Custom Audit data will be available for linkage in centres participating in the NIHR funded NEUROdevelopmental Prognositic after Cardiac Arrest in Kids Trial (NEURO-PACK). This trial will be evaluating more detailed neuro-developmental outcomes of patients after paediatric cardiac arrest.

## References

1. de Caen AR, Maconochie IK, Aickin R, Atkins DL, Biarent D, Guerguerian AM, et al. Part 6: Pediatric Basic Life Support and Pediatric Advanced Life Support: 2015 International Consensus on Cardiopulmonary Resuscitation and Emergency Cardiovascular Care Science With Treatment Recommendations. Circulation. 2015;132(16 Suppl 1):S177-203. Epub 2015/10/17.
2. Moler FW, Silverstein FS, Holubkov R, Slomine BS, Christensen JR, Nadkarni VM, et al. Therapeutic Hypothermia after In-Hospital Cardiac Arrest in Children. N Engl J Med. 2017;376(4):318-29. Epub 2017/01/25.
3. Moler FW, Holubkov R, Dean JM. Therapeutic Hypothermia in Children. N Engl J Med. 2015;373(10):980. Epub 2015/09/04.



For peer review only - http://bmjopen.bmj.com/site/about/guidelines.xhtml

# Data collection method

For units who agree to participate in this custom audit PICANet will enable access to the specific custom audit data collection tab on the data entry page:-

1. A PICANet NET-PACK 3 custom audit form (see below) is completed for all admissions for either out-of-hospital or in-hospital cardiac arrest prior to PICU admission.

**Paediatric Intensive Care Audit Network · Custom Audit** **NET-PACK 3**

*NET-PACK 3: PICANet evaluation of post cardiac arrest care in kids*

*Please complete for **all PIC admissions following cardiac arrest** (include both out-of-hospital and in-hospital arrests)*

**Patient details** *(or hospital label)*

Family name

First name

Postcode

NHS/CHI/H&C number

Case note number

Date of birth *(dd/mm/yyyy)* / /

**History at admission**

*FOR OUT-OF-HOSPITAL CARDIAC ARREST ONLY:*
Bystander CPR attempted?

☐ Yes ☐ No ☐ Unknown

Did CPR continue after arrival to the Emergency Department?

☐ Yes ☐ No ☐ Unknown

*FOR IN AND OUT-OF-HOSPITAL CARDIAC ARREST:*

First monitored cardiac rhythm during cardiac arrest

☐ Asystole
☐ Sinus bradycardia < 60 bpm
☐ Pulseless electrical activity
☐ Ventricular fibrillation
☐ Ventricular tachycardia

*if rhythm detected by ECG*

☐ Shockable
☐ Non-shockable

*if rhythm detected by automated external defibrillator (AED)*

☐ No monitoring
☐ Unknown

Time from observed cardiac arrest to start of sustained return of spontaneous circulation (ROSC)

hours minutes

Number of doses of epinephrine from initial resuscitation to start of period of sustained ROSC

**Temperature management**

Core body temperature management during first 24 hours after sustained ROSC

☐ Active Normothermia *(35 to 37.9 °C)*
☐ Active Therapeutic Hypothermia *(32 to <35 °C)*
☐ Other *(state below)*
☐ No active temperature control
☐ Unknown

Duration of initial active temperature control management *(if temperature actively managed)*

hours

Minimum temperature recorded during first 24 hours

. °C

Maximum temperature recorded during first 24 hours

. °C

**Comments**

**Form completed by**

**Contact us: picanet@leeds.ac.uk**

Jodie Batchelor/Sophie Butler
*Project officer*
(0113) 343 8125
j.a.batchelor@leeds.ac.uk/s.butler1@leeds.ac.uk

Lee Norman
*Database manager*
(0113) 343 8125
l.j.norman@leeds.ac.uk

Caroline Lamming
*Research nurse*
(0116) 252 5414
crl4@leicester.ac.uk

www.picanet.org.uk PICANet custom audit data collection form · NET-PACK 3 · Version 1.3 · April 2017 · Copyright © 2017 Universities of Leeds and Leicester

2. When the PICU enters or uploads to PICANet Web the admission event data for the patient, completion of the PIM field **Cardiac arrest before ICU admission** will permit manual entry of NET-PACK 3 data items.

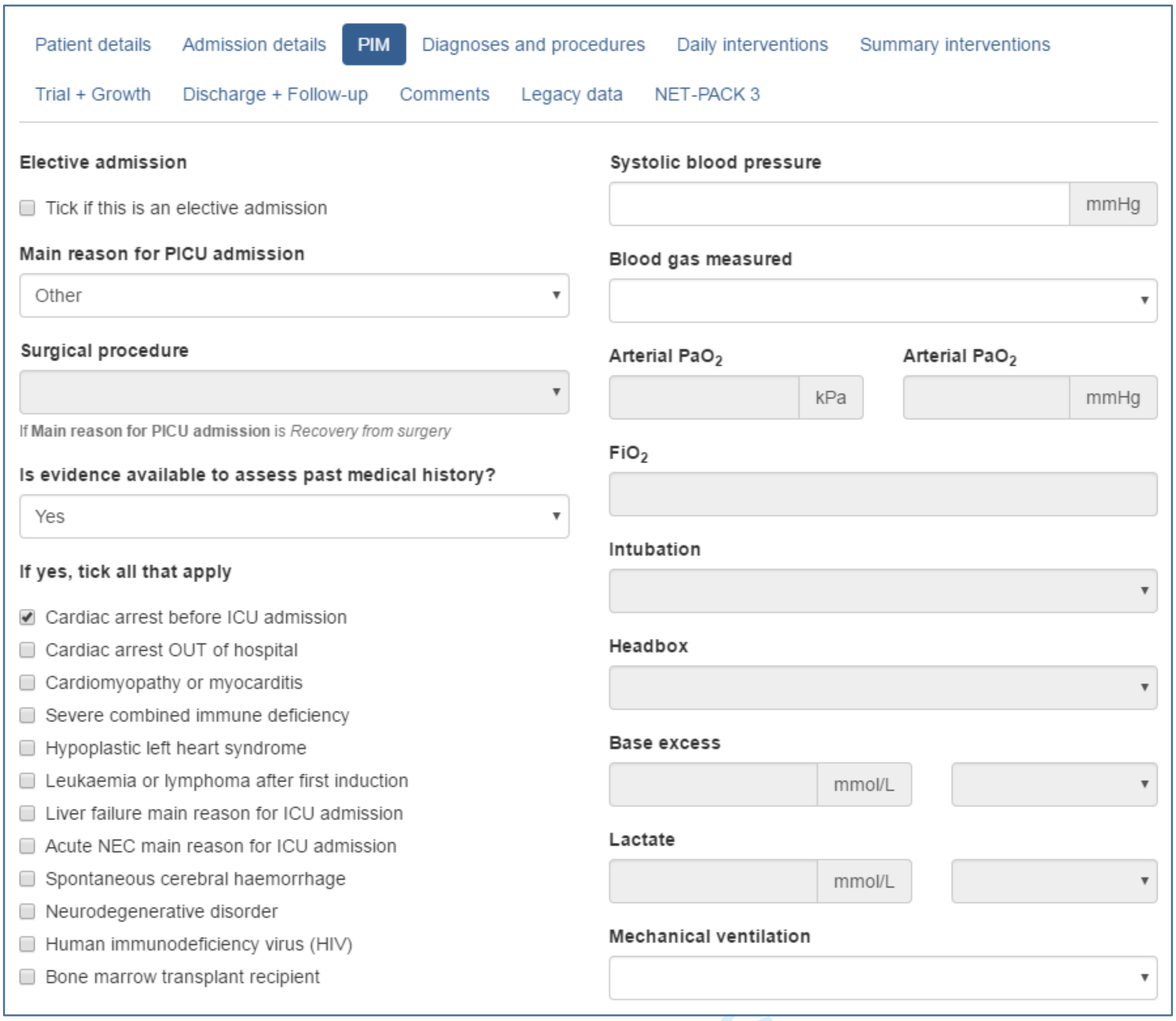

3. To enter NET-PACK 3 data, click the NET-PACK 3 tab. Note that the NET-PACK 3 tab is only visible for applicable events, i.e. when **Cardiac arrest before ICU admission** is ticked.

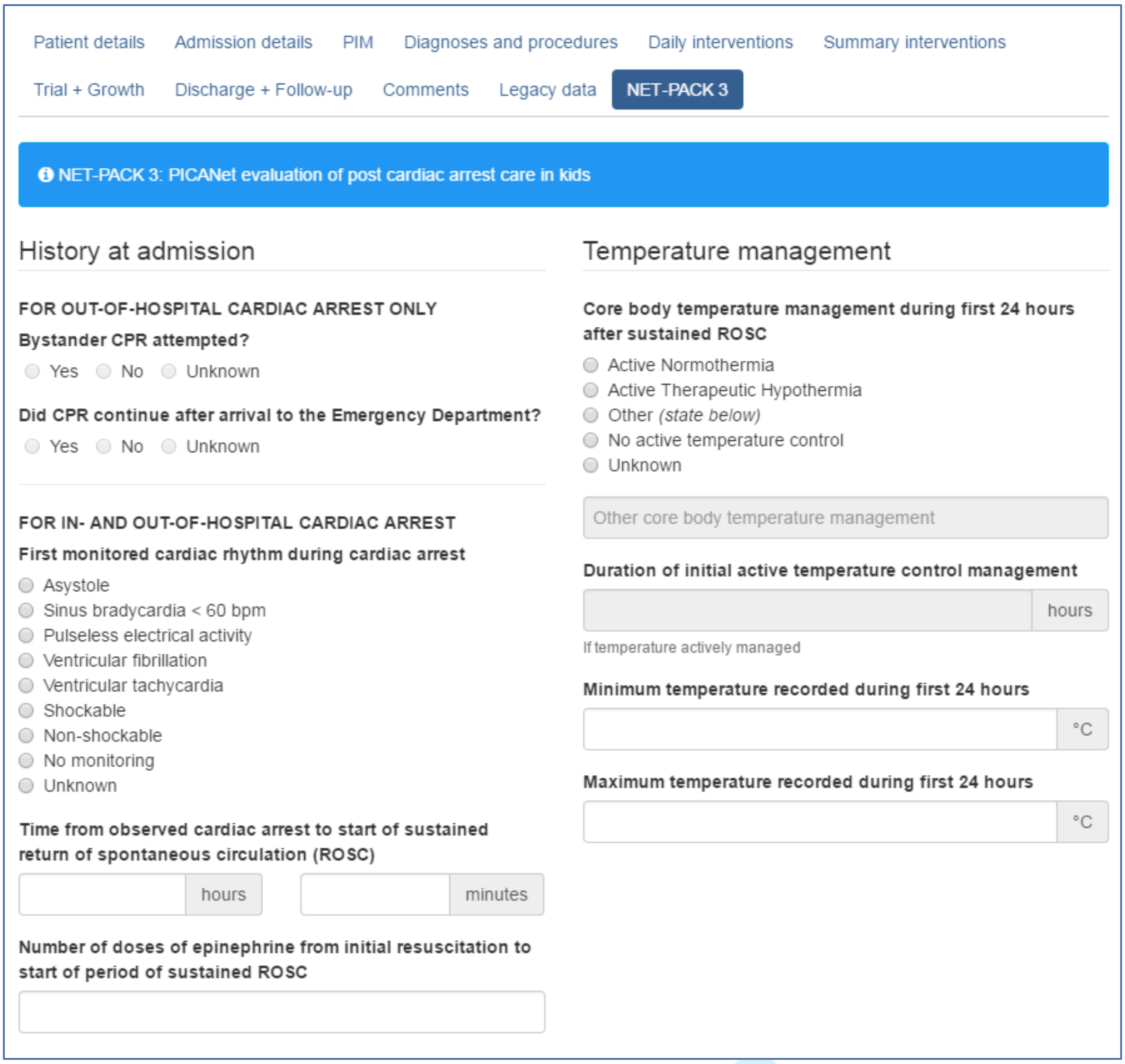
Patient details | Admission details | PIM | Diagnoses and procedures | Daily interventions | Summary interventions
Trial + Growth | Discharge + Follow-up | Comments | Legacy data | NET-PACK 3

NET-PACK 3: PICANet evaluation of post cardiac arrest care in kids

**History at admission**

FOR OUT-OF-HOSPITAL CARDIAC ARREST ONLY

Bystander CPR attempted?
- Yes
- No
- Unknown

Did CPR continue after arrival to the Emergency Department?
- Yes
- No
- Unknown

FOR IN- AND OUT-OF-HOSPITAL CARDIAC ARREST

First monitored cardiac rhythm during cardiac arrest
- Asystole
- Sinus bradycardia < 60 bpm
- Pulseless electrical activity
- Ventricular fibrillation
- Ventricular tachycardia
- Shockable
- Non-shockable
- No monitoring
- Unknown

Time from observed cardiac arrest to start of sustained return of spontaneous circulation (ROSC)
[ ] hours [ ] minutes

Number of doses of epinephrine from initial resuscitation to start of period of sustained ROSC
[ ]

**Temperature management**

Core body temperature management during first 24 hours after sustained ROSC
- Active Normothermia
- Active Therapeutic Hypothermia
- Other *(state below)*
- No active temperature control
- Unknown

Other core body temperature management

Duration of initial active temperature control management
[ ] hours
If temperature actively managed

Minimum temperature recorded during first 24 hours
[ ] °C

Maximum temperature recorded during first 24 hours
[ ] °C

only



For peer review only - http://bmjopen.bmj.com/site/about/guidelines.xhtml

# Patient details

## Family name or Surname

| | |
|---|---|
| **Definition** | The last or family name or surname given to the child as it would appear on the child's birth certificate or other appropriate document. |
| **Reason** | Family name provides an additional identifier that can aid patient tracking throughout the hospital and PICANet Web.<br>Can help identify individuals who may have had multiple admissions to one or more PICUs. |
| **Format** | Free text (e.g. Brown).<br>If no family name available record as UNKNOWN and indicate why not available in the comments section. |

## First name

| | |
|---|---|
| **Definition** | The first name given to the child as it would appear on the child's birth certificate or other appropriate document. |
| **Reason** | First name provides an additional identifier that can aid patient tracking throughout the hospital and PICANet Web.<br>Can help identify individuals who may have had multiple referrals and /or admissions to one or more PICUs. |
| **Format** | Free text (e.g. John).<br>If no first name available record as UNKNOWN and indicate why not available in the comments section. |

## Postcode

| | |
|---|---|
| **Definition** | The postcode for the child's normal place of residence. |
| **Reason** | Postcode provides an additional identifier that can aid patient tracking throughout the hospital and PICANet Web.<br>Can help identify individuals who may have had multiple admissions to one or more PICUs.<br>Postcode provides a means of linkage to geographic and demographic information for effective audit and assessment of health services delivery. |
| **Format** | Text (e.g. S10 8NN).<br>Foreign postcodes will be accepted by the software, although a warning will be generated in the case of non UK standard postcodes to ensure that the user checks the data.<br>If postcode is unobtainable, record as UNKNOWN |



For peer review only

For peer review only - http://bmjopen.bmj.com/site/about/guidelines.xhtml

## NHS, CHI or H&C number

| | |
|---|---|
| **Definition** | Unique identifying number enabling tracing of a patient through the NHS system in England, Wales and Northern Ireland. For English and Welsh patients the NHS number, for Scottish patients the CHI number and for Northern Ireland the H&C number is used as a unique numeric identifier. |
| **Reason** | NHS, CHI or H&C number gives a unique, identifiable variable that will allow other identifiable data items to be removed from the database.<br><br>Can help identify individuals who may have had multiple referrals, transport and/or admission events to one or more PICUs. |
| **Format** | Free text (e.g. 1463788990).<br><br>Validation check that NHS, CHI or H&C number is a valid number |

## Case note number

| | |
|---|---|
| **Definition** | Unique identifying number for an individual's hospital records at the treating unit.<br><br>Allocated on first admission to hospital. |
| **Reason** | Case note number provides a unique identifier that can aid patient tracking throughout the hospital. |
| **Format** | Free text (e.g. AB145C). |

## Date of birth

| | |
|---|---|
| **Definition** | The child's date of birth as recorded on the child's birth certificate or other appropriate document. |
| **Reason** | Date of birth and Date of admission are used to calculate age at admission to your unit.<br><br>Date of birth provides an additional identifier that can aid patient tracking throughout the hospital and PICANet Web.<br><br>Can help identify individuals who may have had multiple referrals and/or admissions to one or more PICUs. |
| **Format** | Date; dd/mm/yyyy.<br><br>Date of birth should be on or prior to the Date of admission.<br><br>If the child's date of birth is unobtainable, but the child is under your care, use your judgement to estimate year of birth and record as 1 January of estimated year (e.g. 01/01/YYYY).<br><br>If information is being extracted from notes and the child's date of birth is not recorded, or recorded as unavailable, leave the field blank and in the 'Indicate if date of birth is' field below tick 'Unknown'.<br><br>If it is necessary for Date of birth to be partly anonymised, enter the correct month and year and record 01 for the day (e.g. 01/MM/YYYY). Then tick 'Anonymised' below. |
| **Validation rule** | Warning if patient is aged 18 years or older |



For peer review only - http://bmjopen.bmj.com/site/about/guidelines.xhtml

# History at admission

## Bystander Cardiopulmonary Resuscitation (CPR) Attempted?

### *For Out-of-Hospital Cardiac Arrest Only*

| | |
|---|---|
| **Definition** | Bystander cardiopulmonary resuscitation (CPR) is CPR performed by a person who is not responding as part of an organized emergency response system approach to a cardiac arrest. Physicians, nurses, and paramedics may be described as performing bystander CPR if they are not part of the emergency response system involved in the victim's resuscitation |
| **Reason** | Recording of this clinical variable can be used to validate a prediction model for hospital survival after out of hospital cardiac arrest. |
| **Format** | Yes<br>No<br>Unknown |
| **Validation rule** | Warning if value not entered |

## Cardiopulmonary Resuscitation continued after arrival to the Emergency Department?

### *For Out-of-Hospital Cardiac Arrest Only*

| | |
|---|---|
| **Definition** | If cardiac arrest and on-going cardiopulmonary resuscitation started in the pre-hospital setting AND continued after arrival in the emergency department record please indicate. |
| **Reason** | Failure to achieve a return of spontaneous circulation (ROSC) in the pre-hospital setting for out of hospital cardiac arrest patients is an important prognostic variable. |
| **Format** | Yes<br>No<br>Unknown |
| **Validation rule** | Warning if value not entered |

For peer review only

## First monitored cardiac rhythm during cardiac arrest

| | |
|---|---|
| **Definition** | Specifies the first cardiac rhythm present when a monitor or defibrillator is attached to a patient during a cardiac arrest.<br>If the automated external defibrillator (AED) does not have a rhythm display, then it may be possible to determine the first monitored rhythm from a data storage card, hard drive, or other device used by the AED to record data.<br>If initial rhythm is detected by an automated electrical defibrillator (AED) with no recording device, record whether the cardiac rhythm was shockable or non-shockable. If there is no ECG monitoring during cardiac arrest, record no monitoring. |
| **Reason** | Recording of this clinical variable can be used to validate a prediction model for hospital survival after out of hospital cardiac arrest. |
| **Format** | If rhythm detected by ECG choose from :<br>Asystole<br>Sinus bradycardia (defined < 60 beats per minute).<br>Pulseless electrical activity,<br>Ventricular fibrillation,<br>Ventricular tachycardia<br>if rhythm detected by an AED without an ECG readout use options:<br>Shockable,<br>Non-shockable<br>if no monitoring during cardiac arrest record<br>No monitoring<br>Unknown |
| **Validation rule** | Warning if value not entered |

## Time from observed cardiac arrest to start of sustained return of spontaneous circulation (ROSC)

| | |
|---|---|
| **Definition** | Time from observed cardiac arrest to start of sustained return of spontaneous circulation (sustained ROSC*) The start time of the cardiac arrest will be the time reported when the child is first identified (found) in cardiac arrest by any bystander e.g. family, public, medical first responder. Estimation of period of time prior to this, which is unwitnessed, will not be included in the duration of cardiac arrest calculation.<br>Sustained Return of Spontaneous Circulation (Sustained ROSC) is deemed to have occurred when chest compressions are not required for 20 consecutive minutes and signs of circulation persist (or Return of circulation by extracorporeal circulatory support, if applied). The 'start' time will be when the initial ROSC (successful resuscitation and the restoration of a spontaneous perfusing rhythm) occurs except where patient has a further cardiac arrest within 20 mins of ROSC. The use of the start time of period of sustained ROSC will therefore take into account multiple cardiac arrests in the initial resuscitation period. |

For peer review only



For peer review only - http://bmjopen.bmj.com/site/about/guidelines.xhtml

**Reason** Duration of cardiac arrest is required to calculate a prediction model for hospital survival after out of hospital cardiac arrest.

**Format** Total number of hours and minutes
[_] hours [__] minutes

**Expected range** 0:01-8:00hrs

**Validation rule** Validation check if time exceeds 8hrs: 00mins
Warning if value not entered

## Number of doses of epinephrine from initial resuscitation to start of period of sustained ROSC

**Definition** Record the total number of individual dose(s) of epinephrine (adrenaline), administered (via any route) from the commencement of initial resuscitation to the start of a period of sustained return of spontaneous circulation greater than 20 minutes (sustained ROSC).

**Reason** An 'Utstein' defined variable required to calculate a prediction model for hospital survival after out of hospital cardiac arrest.

**Format** Numerical value e.g.06

**Expected range** 00 – 40 validation check if number exceeds 40
99 if unknown

**Validation rule** Validation check if number exceeds 40
Warning if value not entered

# Temperature management

## Core body temperature management planned during first 24 hours after sustained ROSC

**Definition** The mode of core body temperature management during the first 24 hours after sustained return of spontaneous circulation (sustained ROSC)

**Active Normothermia** - defined as the active maintenance of core body temperature between 35 and <38 degrees Celsius)

**Active Therapeutic Hypothermia** - defined as active reduction of core body temperature to between 32 to <35 degrees Celsius)

**Other -** (complete comments box)

**No active temperature control**

**Unknown**

**Reason** An 'Utstein' defined variable required to calculate a prediction model for hospital survival after out of hospital cardiac arrest.



For peer review only - http://bmjopen.bmj.com/site/about/guidelines.xhtml

| | |
|---|---|
| **Format** | Choose from one of the following:<br>Active Normothermia<br>Active Therapeutic hypothermia -<br>Other - complete text box<br>No active temperature control<br>Unknown |
| **Validation rule** | Warning if value not entered |

## Duration of initial active temperature control management

| | |
|---|---|
| **Definition** | The duration of active temperature management if the core body temperature is actively managed by normothermia, therapeutic hypothermia or other stated method. |
| **Reason** | Required to provide further detail about active core body temperature processes |
| **Format** | Insert the total number of hours e.g.24 hours<br>if unknown insert 999 |
| **Expected range** | 1 – 120 hrs. |
| **Validation rule** | Validation check if number exceeds 120<br>Warning if temperature management type = Normothermia, Therapeutic hypothermia or other and no value added |

## Minimum temperature recorded during first 24 hours

| | |
|---|---|
| **Definition** | The minimum temperature recorded during the first 24 hours after start of sustained return of spontaneous circulation (sustained ROSC). |
| **Reason** | Required to provide further detail about active core body temperature processes. |
| **Format** | Record in degrees Celsius e.g. 32.5 °C<br>if unknown record 999 |
| **Expected range** | 20.00-42 00 °C |
| **Validation rule** | Validation check if number exceeds 42.00 °C<br>Add warning if value not entered |

## Maximum temperature recorded during first 24 hours

| | |
|---|---|
| **Definition** | The maximum temperature recorded during the first 24hours after start of sustained return of spontaneous circulation (sustained ROSC). |
| **Reason** | Required to provide further detail about active core body temperature processes. |



For peer review only - http://bmjopen.bmj.com/site/about/guidelines.xhtml

**Format** Record in degrees Celsius e.g. 37.5°C
if unknown record 999

**Expected range** 20.00-42.00°C

**Validation rule** Validation check if number exceeds 42.00 °C
Add warning if value not entered
Add warning if maximum temperature <= minimum temperature

## Comments

**Definition** Any additional information considered relevant to the dataset.

Text entered in this field may provide extra information about data entered elsewhere in a specific field in the dataset, or may provide extra information on the admission, which is not collected as part of the dataset.

No identifiers (patient, nurse, doctor, ICU, hospital) should be included in text data entered into this field.

As there is limited space in this field all text data should be kept to a minimum and be as concise as possible. Text data must not contain any punctuation except a period (full-stop) at the end of each data point.

**Reason** No dataset specification covers all eventualities: to deal with this a text field has been included for comments/additional information.

**Format** Free text

## Form completed by

**Definition** Name of person completing form.

**Reason** For local use only to assist with following up queries relating to completion of this form.

**Format** Free text



For peer review only

For peer review only - http://bmjopen.bmj.com/site/about/guidelines.xhtml

*NET-PACK 3: PICANet evaluation of post cardiac arrest care in kids*

*Please complete for* ***all PIC admissions following cardiac arrest*** *(include both out-of-hospital and in-hospital arrests)*

## Patient details *(or hospital label)*

**Family name**

☐

**First name**

☐

**Postcode**

☐☐☐☐ ☐☐☐

**NHS/CHI/H&C number**

☐☐☐ ☐☐☐ ☐☐☐☐

**Case note number**

☐☐☐☐☐☐☐☐☐☐

**Date of birth** *(dd/mm/yyyy)*

☐☐ / ☐☐ / ☐☐☐☐

## History at admission

***FOR OUT-OF-HOSPITAL CARDIAC ARREST ONLY:***

**Bystander CPR attempted?**

☐ Yes ☐ No ☐ Unknown

**Did CPR continue <u>after</u> arrival to the Emergency Department?**

☐ Yes ☐ No ☐ Unknown

***FOR IN AND OUT-OF-HOSPITAL CARDIAC ARREST:***

**First monitored cardiac rhythm during cardiac arrest**

- ☐ Asystole
- ☐ Sinus bradycardia < 60 bpm
- ☐ Pulseless electrical activity
- ☐ Ventricular fibrillation
- ☐ Ventricular tachycardia

*} if rhythm detected by ECG*

- ☐ Shockable
- ☐ Non-shockable

*} if rhythm detected by automated external defibrillator (AED)*

- ☐ No monitoring
- ☐ Unknown

**Time from observed cardiac arrest to start of sustained return of spontaneous circulation (ROSC)**

☐ hours ☐☐ minutes

**Number of doses of epinephrine from initial resuscitation to start of period of sustained ROSC**

☐☐

## Temperature management

**Core body temperature management during first 24 hours after sustained ROSC**

- ☐ Active Normothermia *(35 to 37.9 °C)*
- ☐ Active Therapeutic Hypothermia *(32 to <35 °C)*
- ☐ Other *(state below)*
- ☐ No active temperature control
- ☐ Unknown

☐

**Duration of initial active temperature control management** *(if temperature actively managed)*

☐☐ hours

**Minimum temperature recorded during first 24 hours**

☐☐ . ☐ °C

**Maximum temperature recorded during first 24 hours**

☐☐ . ☐ °C

## Comments

☐

## Form completed by

☐

## Contact us: picanet@leeds.ac.uk

**Sophie Butler**
*Project officer*
(0113) 343 8125
s.butler1@leeds.ac.uk

**Lee Norman**
*Database manager*
(0113) 343 8125
l.j.norman@leeds.ac.uk

**Caroline Lamming**
*Research nurse*
(0116) 252 5414
crl4@leicester.ac.uk

For peer review only - http://bmjopen.bmj.com/site/about/guidelines.xhtml
